# Mammalian ZAP and KHNYN independently restrict CpG-enriched avian viruses

**DOI:** 10.1101/2024.12.23.629495

**Published:** 2024-12-23

**Authors:** Jordan T. Becker, Clayton K. Mickelson, Lauren M. Pross, Autumn E. Sanders, Esther R. Vogt, Frances K. Shepherd, Chloe Wick, Alison J. Barkhymer, Stephanie L. Aron, Elizabeth J. Fay, Reuben S. Harris, Ryan A. Langlois

**Affiliations:** 1 Department of Microbiology and Immunology, University of Minnesota – Twin Cities, Minneapolis, MN, USA, 55455; 2 Department of Biochemistry, Molecular Biology, and Biophysics, University of Minnesota – Twin Cities, Minneapolis, MN, USA, 55455; 3 Department of Biochemistry and Structural Biology, University of Texas Health, San Antonio, TX, USA, 78229; 4 Howard Hughes Medical Institute, University of Texas Health, San Antonio, TX, USA, 78229; 5 Co-corresponding authors; 6 Lead contact

**Keywords:** ZAP, KHNYN, influenza A virus, Rous sarcoma virus, host-pathogen interactions, BIOLOGICAL SCIENCES, MICROBIOLOGY

## Abstract

Zoonotic viruses are an omnipresent threat to global health. Influenza A virus (IAV) transmits between birds, livestock, and humans. Proviral host factors involved in the cross-species interface are well known. Less is known about antiviral mechanisms that suppress IAV zoonoses. We observed CpG dinucleotide depletion in human IAV relative to avian IAV. Notably, human ZAP selectively depletes CpG-enriched viral RNAs with its cofactor KHNYN. ZAP is conserved in tetrapods but we uncovered that avian species lack KHNYN. We found that chicken ZAP does not affect IAV (PR8) or CpG enriched IAV. Human ZAP or KHNYN independently restricted CpG enriched IAV by overexpression in chicken cells or knockout in human cells. Additionally, mammalian ZAP-L and KHNYN also independently restricted an avian retrovirus (ROSV). Curiously, platypus KHNYN, the most divergent from eutherian mammals, was also capable of direct restriction of multiple diverse viruses. We suggest that mammalian KHNYN may be a *bona fide* restriction factor with cell-autonomous activity. Furthermore, we speculate that through repeated contact between avian viruses and mammalian hosts, protein changes may accompany CpG-biased mutations or reassortment to evade mammalian ZAP and KHNYN.

## INTRODUCTION

Influenza A virus (IAV) is a constantly circulating infectious burden in global waterfowl populations, agricultural animals, and among humans seasonally. Avian IAV occasionally transmits into domesticated mammals and humans. While these infections do not usually result in successful human-to-human spread, they are distinctly pathogenic with a reportedly high mortality rate. However, when novel avian IAV establishes in the human population there can be devastating consequences. Major pandemics have been the result of avian-to-human zoonoses or mixed-animal zoonoses involving avian-swine-human infection chains(1). Notably, multiple pandemics or large-scale epidemics occurred in 1918, 1957, 1968, and 2009 that resulted in massive infection incidence and mortality in human populations(2). Much is known about the host dependency factors to which avian IAV must adapt to infect human cells productively(3). These include differences in viral hemagglutinin binding to host sialic acid linkages(4) with species-specific airway localization(5), pH-sensitivity of viral RNA (vRNA) endosomal release into the cytoplasm(6), and species-specific ability to use the polymerase cofactor, ANP32(7). Relatively less is known about the network of restriction factors that might specifically target zoonotic IAV infections in mammals(3). Much of our knowledge of host-pathogen interactions and genetic conflicts has focused on amino acid level and related protein structure changes that predicate animal-to-human adaptation(8). Comparatively less is known about nucleic acid adaptations driven by host-pathogen conflicts during interspecies infections.

Viral nucleic acid sequence and structure are not only a determinant of protein coding changes, but rather they encompass a complex and sensitive network of features that determine viral fitness, stability, and pathogenesis(9, 10). In addition, host and pathogen genomes are not in equilibrium regarding the frequency and usage of nucleotide doublets(11, 12). For example, CpG dinucleotide frequency is depleted throughout the human genome(13). Commonly, this is a result of DNA methylation at CpG motifs near promoter sequences that regulate transcription(14). Mammalian genomes have also selected against encoding CpG dinucleotides in cytoplasmic-resident RNAs(13, 15). Others observed a depletion of CpG content among interferon stimulated genes (ISGs), notably in mammalian species(13, 16). Accordingly, many viral pathogens that infect mammalian species match the dinucleotide content of their host species and mammalian viruses are often CpG-depleted(11, 13, 17, 18). Others showed that avian IAV sequences exhibit elevated CpG content and that CpG content has depleted over time for IAV strains circulating in humans(13, 18). This suggests that host factors might exist in human hosts to select against CpG-enriched viral nucleic acids that are absent or defective in avian species.

Recently, several groups showed that the human restriction factor, zinc-finger antiviral protein (ZAP also known as PARP13), specifically binds to and suppresses CpG dinucleotides in cytoplasmic RNA(19, 20). Human ZAP is encoded by the *ZC3HAV1* gene, induced by interferon, and generates multiple isoforms(21, 22), including the ZAP-L (a 902 amino acid isoform preferentially localized to intracellular membranes) and ZAP-S (a 699 amino acid isoform with diffuse cytoplasmic localization). Like many antiviral proteins, ZAP is evolving under positive selection(23–25). ZAP antiviral activity is regulated by the interferon-inducible E3 ubiquitin ligase, TRIM25(19, 20, 26, 27). KHNYN was identified as a ZAP interacting protein and putative cofactor for ZAP antiviral activity(20). Moreover, KHNYN exhibits basal expression and is not interferon-inducible(28). While KHNYN can exert antiviral effects through ZAP it is unknown if it can exert additional direct antiviral functions. Together, human ZAP, KHNYN, and TRIM25 may function cooperatively, selectively, and/or independently to restrict certain classes of viruses with a bias towards viruses with CpG-enriched nucleotide content. While the function of these human proteins has been well studied, less is known about their non-primate and non-mammalian orthologues and their antiviral restriction ability.

Understanding the intrinsic and innate immune factors capable of restricting zoonotic viruses is paramount in identifying zoonotic viruses with pandemic potential(29). Importantly, IAV is a seasonal infectious burden in humans and common zoonosis among wild waterfowl, agricultural birds, domesticated mammals, and humans. We hypothesized that birds are defective in RNA recognition and restriction pathways and that presents a barrier to emergence in mammals. Here, we tested whether restriction factors targeting CpG-enriched RNAs could affect replication of IAV with an elevated CpG-content, mimicking avian IAV. We found that chicken and duck ZAP do not restrict IAV replication regardless of CpG content. We show through genomic, phylogenetic, and functional analyses that avian species are defective in ZAP function. Additionally, we demonstrate that avian species lack KHNYN, which likely arose through gene duplication after birds and mammals diverged. Thus, we used chicken cells as a gain-of-restriction system to test the exogenous provision of host factors for IAV restriction. Indeed, human ZAP-S and KHNYN stably expressed in chicken cells independently restricted CpG-enriched IAV replication. We showed that combined knockout of ZAP and KHNYN in human cells increased replication of CpG-enriched IAV to a greater extent than single knockout of ZAP or KHNYN alone. We also found that mammalian but not avian (*Galloanserae*) ZAP as well as some mammalian KHNYN proteins restrict a naturally CpG-enriched avian retrovirus, Rous sarcoma virus (ROSV). Unexpectedly, we discovered a potently antiviral KHNYN homologue from platypus that suggests an ancient restriction mechanism. We show that KHNYN is a *bona fide* restriction factor with cell-autonomous activity. Furthermore, we speculate that through repeated contact between avian and human hosts followed by seasonal circulation among humans, protein level changes for receptor preference and polymerase function may be accompanied by dinucleotide changes and/or CpG-disfavored reassortment that evade restriction by mammalian ZAP and KHNYN.

## RESULTS

### Mammalian IAV sequences are CpG-depleted relative to avian IAV.

**Figure 1:**
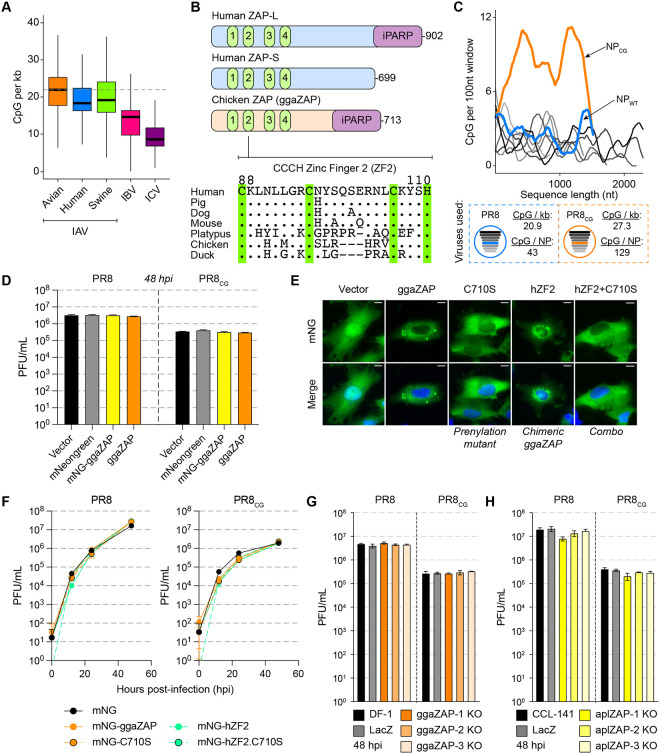
Avian ZAP does not restrict replication of CpG-enriched IAV. **(A)** IAV sequences exhibit depletion of CpG content (CpG per kb = # CpG / segment length in kilobases [kb]). Box and whisker plots showing CpG content of all IAV sequences from avian (orange), human (blue), and swine (green) hosts as well as IBV (pink) and ICV (purple). Thick black bars represent median CpG content, boxes define the 25^th^ to 75^th^ percentile, and whiskers represent range excluding outliers. Horizontal dashed gray lines highlight median avian IAV CpG content as a reference. **(B)** Schematic of human ZAP-L, human ZAP-S and chicken ZAP (ggaZAP) protein domain architecture including four CCCH zinc fingers and inactive poly-ADP ribose polymerase (iPARP). Amino acid alignment of ZAP zinc finger 2 (ZF2; human residues 88–110) from multiple species. Conserved CCCH residues are highlighted with green vertical bars. **(C)** CpG content of PR8 (shades of gray by segment length), wild-type NP (blue), and CpG-enriched NP (orange) calculated as # CpG per 100 nucleotides (nt) over a sliding window (top). Schematic of PR8 and PR8_CG_ viruses and displaying CpG content for each virus and total CpG number encoded by each NP segment (bottom). **(D)** Titers of PR8 and PR8_CG_ at 48 hours post infection (hpi) in chicken DF-1 cells stably expressing mNeonGreen (mNG, gray), chicken ZAP (ggaZAP, yellow), or mNG-ggaZAP (orange) as well as control transduced cells (Vector, black). Bar represents mean plaque forming units per mL (PFU/mL) with error bars representing standard deviation of the mean from three biological replicates. Multiplicity of infection (MOI) = 0.05 (pfu per cell). **(E)** Fluorescence images of DF-1 cells expressing mNG as well as fusion proteins of mNG-ggaZAP and mutants indicated (green, top). Nuclei counterstained with DAPI (blue) and shown in merge image (bottom). Scale bars represent 10 microns. **(F)** Titers of PR8 and PR8_CG_ over 48 hpi in chicken DF-1 cells stably expressing mNG-ggaZAP mutants shown in (E). MOI=0.05. **(G)** Titers of PR8 and PR8_CG_ at 48 hpi in parental untransduced DF-1 cells (DF-1, black), pooled CRISPR/Cas9 knockout chicken DF-1 cells expressing a control sgRNA targeting E. coli LacZ (LacZ, gray), or sgRNAs targeting endogenous chicken ZC3HAV1 (ggaZAP1–3 KO, shades of orange). MOI=0.05. **(H)** Titers of PR8 and PR8_CG_ at 48 hpi in parental untransduced CCL-141 cells (CCL-141, black), pooled CRISPR/Cas9 knockout duck CCL-141 cells expressing a control sgRNA targeting E. coli LacZ (LacZ, gray), or sgRNAs targeting endogenous duck ZC3HAV1 (aplZAP1–3 KO, shades of yellow). MOI=0.05.

Viruses evolve to replicate successfully in their host species. Previous work showed that human IAV exhibited a depletion of CpG dinucleotide content relative to avian IAV sequences since the 1918 IAV pandemic(13, 18, 30). Here, surveying influenza sequences in FluDB(31) collected between 1918 and 2020, we also observed a depletion of CpG dinucleotides per kilobase (CpG content) in human and swine but not avian IAV sequences over time since 1918 (Figure S1A). By comparing all human, swine, and avian IAV segment sequences *en masse*, we observed a lower CpG content in human and swine IAV relative to avian IAV sequences (Figure 1A). We observed similar results for ρ_CG_ (rho accounts for total C and G content(12, 13, 16)) but less so for other dinucleotides or %GC content (Figure S2A–D). Notably, the largely human-specific influenza B virus(32) (IBV) and influenza C virus(33) (ICV) are CpG-depleted relative to all IAV sequences, speculatively consistent with long-term near-exclusive circulation in mammalian/human individuals (Figure 1A, S1, and S2). We found that when comparing major HA and NA subtypes, CpG content is depleted in subtypes seasonally circulating in humans (H1N1 and H3N2) relative to avian sequences of the same subtype (Figure S1C). Conversely, CpG content in human isolates of common avian-to-human zoonotic HA and NA subtypes (e.g. H5N1 and H7N9) is similar to their avian counterparts. We observed that human IAV segments 1–3 encoding the heterotrimeric viral polymerase (i.e. PB2, PB1, and PA) are more CpG-depleted relative to avian IAV than segments 4–6 encoding HA, NP, and NA, virion proteins typically subject to adaptive immune pressure(34) (Figure S1B). The natural history of human IAV includes multiple introductions(35) resulting from avian zoonoses and/or reassortment of avian IAV segments with human viruses in 1918, 1957, 1968, and 2009. We compared CpG content of avian IAV to human IAV during the time periods surrounding the replacement of H1N1 with H2N2 in 1957, H2N2 with H3N2 in 1968, reintroduction of H1N1 in 1977, and pandemic “swine flu” H1N1 in 2009 (Figure S1D, bottom). We observed that CpG content is lower in human relative to avian IAV sequences during the longer time periods following zoonoses in 1977 and 2009 (Figure S1D, top). This may reflect periodic reintroduction of CpG-enriched avian sequences that deplete over longer time frames.

### Avian ZAP does not restrict replication of CpG-enriched IAV.

We hypothesized that avian species are defective in cytoplasmic CpG-sensing by ZAP and reasoned that this deficiency allows CpG-maintenance of IAV sequences in avian host species. Human ZAP-L and chicken ZAP are homologous proteins encoding four zinc fingers, an inactive poly-ADP-ribose polymerase-like domain(36), and carboxy-terminal prenylation motif(22, 37) (Figure 1B, top). Previous structural and functional analysis of human ZAP identified zinc finger 2 (ZF2, residues 88–110) as responsible for directly recognizing and binding to CpG dinucleotides in the context of short (3–20 nucleotide) and long (full-length 9 kilobase HIV-1 genome) RNA molecules(19, 38, 39). Using two structural prediction algorithms, we generated models of the four zinc fingers of chicken ZAP (ggaZAP, residues 1–220) by both comparative modeling based on human ZAP (Robetta-CM(40), PDB: 6UEJ(38)) and *ab initio* folding by RoseTTAFold(41) (Figure S3A–B). We also compared these structures to the model of chicken ZAP predicted by AlphaFold2(42) (Figure S3A–B). As expected, we observed that both *ab initio* predicted structures (RoseTTAFold and AlphaFold2) exhibited lower structural similarity (as measured by root mean squared deviation, RMSD) to human ZAP relative to those generated with comparative modeling (Figure S3B). In addition, we observed structural differences between human ZAP and models of duck ZAP as well as quail ZAP (Figure S3A–B). Alignment of the amino acid sequences of ZAP homologues revealed multiple amino acid differences between ZF2 of avian and mammalian species (Figure 1B). Therefore, we hypothesized that chicken ZAP is unable to restrict CpG-enriched viruses and tested this hypothesis using the common IAV laboratory strain A/Puerto Rico/8/1934//H1N1 (PR8) and a CpG-enriched version of PR8(43) (PR8_CG_, orange dashed box; Figure 1C).

In chicken DF-1 cells stably expressing chicken ZAP (ggaZAP) as well as mNeonGreen-tagged chicken ZAP (mNG-ggaZAP), we found no difference in virus replication over 48 hours for PR8 or PR8_CG_ relative to vector-transduced (Vector) and mNeonGreen-transduced (mNG) cells (Figure 1D). The chicken *ZC3HAV1* gene encodes a single ZAP isoform of 713 amino acids with a carboxy-terminal prenylation motif (amino acids: _710_CIVC_713_) that is homologous to the long isoform of human ZAP (ZAP-L). The short isoform of human ZAP (ZAP-S) is generated by alternative splicing and lacks the carboxy-terminal prenylation motif and the inactive poly-ADP-ribose polymerase-like domain. To test if either prenylation of chicken ZAP or if the divergent ZF2 regulate restriction of CpG-enriched IAV, we generated mutants of chicken ZAP lacking the critical cysteine residue (C710S) for membrane-targeting prenylation, a chimera encoding the human ZF2 (hZF2), or a combination (hZF2.C710S) of these two mutants all of which expressed and localized as expected (Figure 1E). However, no form of chicken ZAP restricted PR8 or PR8_CG_ replication (Figure 1F). Finally, we found that pooled CRISPR/Cas9-mediated knockout of endogenous ZAP in chicken DF-1 and duck CCL-141 cells had no effect on PR8 or PR8_CG_ replication (Figure 1G–H and S4). Similarly, we observed that knockdown of endogenous ZAP by stable shRNA in chicken DF-1 cells or transient siRNA in duck CCL-141 cells had no effect on PR8 or PR8_CG_ replication (Figure S3C–E). qPCR analysis showed endogenous chicken ZAP is expressed in DF-1 cells, induced by interferon, and can be suppressed by shRNA (Figure S3C). Given that human ZAP can use chicken TRIM25 as a cofactor(44), we reasoned that chicken DF-1 cells are deficient in ZAP-dependent antiviral activity targeting this CpG-enriched IAV and could serve as a gain-of-restriction system to test other host factors for the ability to restrict CpG-enriched viruses.

### Human ZAP-S and KHNYN independently restrict CpG-enriched IAV.

**Figure 2:**
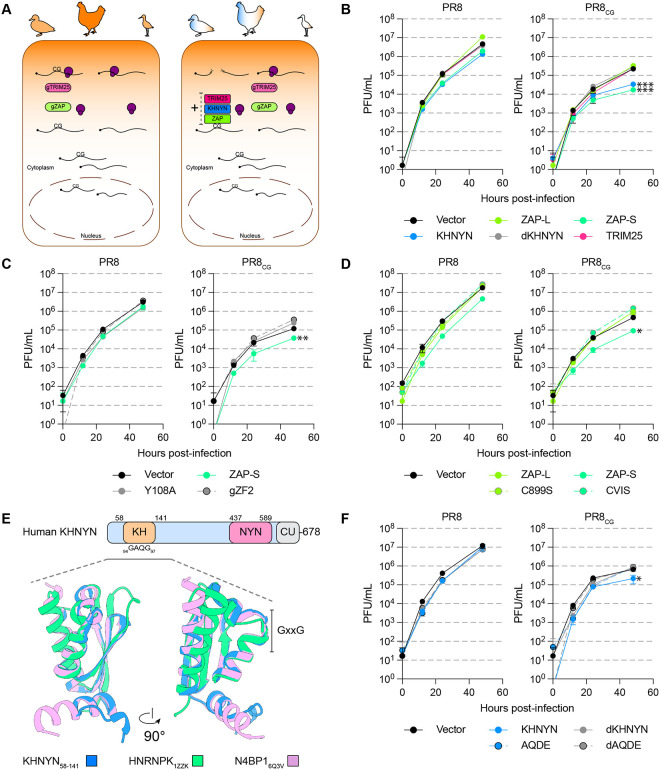
Human ZAP-S and KHNYN independently restrict CpG-enriched IAV. **(A)** Schematic showing lack of CpG-targeting activity in chicken cells (left) and restriction of CpG-enriched cytoplasmic RNAs by human ZAP, KHNYN, or TRIM25 transduced into chicken cells (right). **(B)** Titers of PR8 and PR8_CG_ over 48 hpi in chicken DF-1 cells stably expressing human ZAP-L (green), ZAP-S (lime green), KHNYN (blue), dKHNYN (gray), or TRIM25 (pink) relative to Vector (black). MOI=0.05. *** indicates p≤0.0001 at 48hpi by two-way ANOVA. **(C)** Titers of PR8 and PR8_CG_ over 48 hpi in chicken DF-1 cells stably expressing RNA-binding mutants of ZAP-S (lime green) including Y108A (gray with solid line) and chimeric encoding chicken ZF2 (gZF2; gray with black outline and dashed line) relative to vector (black). MOI=0.05. ** indicates p=0.0093 at 48hpi by two-way ANOVA. **(D)** Titers of PR8 and PR8_CG_ over 48 hpi in chicken DF-1 cells stably expressing C-terminal mutants of human ZAP-L (green) and ZAP-S (lime green) including ZAP-L C899S (green with gray outline and dashed line) and ZAP-S +CVIS motif (CVIS; lime green with gray outline and dashed line). MOI=0.05. * indicates p=0.0108 at 48hpi by two-way ANOVA. **(E)** Schematic of human KHNYN protein showing KH-like domain (tan), NYN endonuclease domain (pink), and CUBAN domain (gray) as well as the GXXG motif with amino acid locations indicated (top). Structure and model of human KH-domains including KHNYN (residues 58–141, blue, RobettaCM model), HNRNPK (green, PDB: 1ZZK), and N4BP1 (pink, PDB: 6Q3V) from two views (bottom). Putative GXXG motif is indicated at top of the central alpha-helix of the three structures. **(F)** Titers of PR8 and PR8_CG_ over 48 hpi in chicken DF-1 cells stably expressing mutants of KHNYN including catalytically inactive dKHNYN (gray), _95_AQ/DE_96_ mutant (AQDE; blue dot with black outline and light blue dashed line), and combination mutant of dKHNYN with AQDE (dAQDE; gray dot with black outline and gray dashed line). MOI=0.05. * indicates p=0.0158 at 48hpi by two-way ANOVA.

Restriction of CpG RNA has been attributed to human ZAP along with its cofactors KHNYN and/or TRIM25. However, we found that avian species lacked a *bona fide* KHNYN orthologue. There are no annotated avian KHNYN orthologues in ensembl, NCBI, or UCSC Genome Browser and a ~400,000bp syntenic region surrounding human KHNYN is missing in avian species, including the KHNYN paralogue – NYNRIN (Figure S5A). Human KHNYN and NYNRIN are encoded in tandem on chromosome 14 and NYNRIN is the result of a KHNYN gene duplication and fusion to a retroviral integrase domain(45) (Figure S5B). KHNYN and NYNRIN are paralogous to N4BP1(46), a gene conserved in birds, reptiles, and fish (Figure S5B–C). We estimated a maximum likelihood phylogeny of human KHNYN, NYNRIN, and N4BP1 orthologues nucleotide sequences from ensembl (Figure S5C). We confirmed that N4BP1 is conserved in *Gnathostomats* (i.e. jawed vertebrates including fish, birds, reptiles, and mammals) and is closely related to a fish-specific N4BP1 paralogue (sometimes annotated as *khnyn* or *khnyn-like*, e.g. *Danio rerio khnyn*, ENSDARG00000092488). However, KHNYN found in mammalian species (i.e. monotremes, marsupials, and placentals) and NYNRIN found in therian mammals (i.e. marsupials and placentals) branched together and are more like each other than to the N4BP1 genes (Figure S5C). We noted that NCBI and ensembl contain reptile sequences annotated as *KHNYN* (e.g. *Alligator mississippiensis KHNYN* XM_014607035.3 or *Anolis carolinensis khnyn* ENSACAG00000009105) that may represent ancestral KHNYN orthologues based on nucleotide sequence (Figure S5C; ancient KHNYN orthologues), exon organization, and syntenic neighborhood (Figure S5D). Thus, we reasoned that avian species are defective and/or deficient in two restriction factor genes: ZAP as well as KHNYN. As such, we generated chicken DF-1 cells that stably expressed human ZAP-L, ZAP-S, KHNYN, or TRIM25 (schematic in Figure 2A). We included a mutant of KHNYN that is expected to be enzymatically inactive within its endonuclease domain(20) due to substitution of three critical aspartic acid residues (DDD443/524/525AAA, dKHNYN). We found that ZAP-S as well as KHNYN modestly restricted PR8 relative to vector transduced cells (Figure 2B, left). KHNYN has not been shown previously to act independently of ZAP. Surprisingly, we found that human ZAP-S and KHNYN (but not ZAP-L, dKHNYN, or TRIM25) independently restricted PR8_CG_ relative to vector (Figure 2B, right).

### CpG-targeted restriction requires intact RNA-binding domains of ZAP and KHNYN.

To confirm the role of ZAP-S and KHNYN in restricting PR8_CG_ we generated mutants of each, stably expressed them in DF-1 cells, and performed multicycle IAV replication assays. To test the CpG specificity of ZAP-S in restricting PR8_CG_, we generated a Y108A mutant previously shown to reduce CpG specificity(38) as well as a chimeric human ZAP-S encoding the ZF2 of chicken ZAP (gZF2). Only WT human ZAP-S remained capable of restricting PR8_CG_, an ability that was lost by the Y108A and gZF2 mutants (Figure 2C). To test if the prenylation motif (amino acids: CVIS) and corresponding membrane localization of ZAP regulated restriction activity against PR8_CG_, we generated a mutant of ZAP-S that encoded a carboxy-terminal CVIS motif (+CVIS) as well as a C899S mutant of ZAP-L(22). While the C899S mutant of human ZAP-L did not gain the ability to restrict PR8_CG_, human ZAP-S +CVIS lost the ability to restrict PR8_CG_ as expected (Figure 2D). Next, to delineate the manner of KHNYN restriction of PR8_CG_ we mutated its KH domain. KH-domain containing proteins encode a GXXG motif at the top of a central alpha-helix and mutations in the GXXG motif reduced RNA-interactions in another KH-domain containing protein, HNRNPK(47). We generated models of the KH domain of KHNYN using comparative modeling based on the third KH-domain of human HNRNPK(48) (Robetta-CM; PDB: 1ZZK) as well as by *ab initio* folding (RoseTTAFold) (Figure 2E; Figure S5E). Both models showed a central alpha-helix with the _94_GAQG_97_ motif at its apex adjacent to a three-helix bundle (Figure S5E). We found that only WT human KHNYN was capable of restricting PR8_CG_ over 48 hours of replication in chicken DF-1 cells but not the catalytically inactive dKHNYN, a AQ95/96DE mutant (AQDE), or a catalytically inactive AQ95/96DE (dAQDE) (Figure 2F). Together, these results indicated that intact human ZAP-S as well as KHNYN can independently restrict PR8_CG_.

### ZAP-S and KHNYN do not alter chicken or IAV mRNA.

To understand how these mammalian restriction factors functionally inhibit IAV, we examined transcriptomic changes during IAV infection. We performed RNA-seq on uninfected as well as PR8 or PR8_CG_ infected chicken DF-1 cells stably expressing human ZAP-L, ZAP-S, KHNYN, or dKHNYN to evaluate if these human proteins modulated the chicken transcriptome and viral mRNA expression relative to vector transduced cells. We found that the transcriptomes of uninfected cells were largely unperturbed by whatever human factor was overexpressed (Figure S6A) suggesting antiviral activity may not be caused by changing the underlying gene expression profile. Next, we hypothesized that by interacting with and depleting CpG-enriched viral RNAs during multicycle IAV replication experiments, ZAP-S and KHNYN might also negatively bias CpG-enriched segment accumulation during replication. We evaluated relative levels of IAV RNA segments in PR8 versus PR8_CG_ infected cells in the presence of these different human factors and found similar levels of viral mRNA within segments (Figure S6B). Infection with either PR8 or PR8_CG_ resulted in similar gene expression patterns for host cell factors between the two viruses, suggesting that the mechanism of restriction is not through differential induction of host antiviral responses (Figure S6C). Together these results suggested initial mechanistic insights into how ZAP and KHNYN might restrict IAV during zoonotic infections although the restriction may not directly target/deplete viral mRNAs.

### Endogenous human ZAP and KHNYN restrict CpG-enriched viruses.

**Figure 3:**
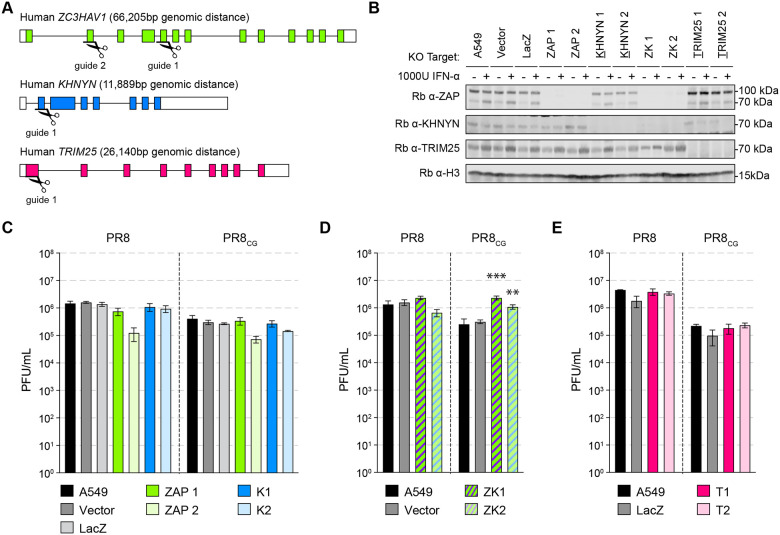
Endogenous human ZAP and KHNYN independently restrict CpG-enriched IAV. **(A)** Schematic depicting exon organization of human ZAP (green), KHNYN (blue), and TRIM25 (pink) genes and sites targeted by CRISPR single guide RNAs (sgRNAs; scissors). Colored boxes indicate coding exons and white boxes indicate noncoding portions of exons. **(B)** Western blots of CRISPR/Cas9 engineered knockout (KO) human A549 cell lines including parental A549 cells, control clone transduced with pLentiCRISPR1000 (Vector), control clone targeting E. coli LacZ (LacZ), two ZAP KO clones (ZAP1 and ZAP2), two KHNYN KO clones (K1 and K2), two ZAP and KHNYN dual KO clones (ZK1 and ZK2), and two TRIM25 KO clones (T1 and T2). Cells were treated with vehicle (PBS) or 1000 units per mL (U/mL) universal interferon-alpha (IFNα) for 24 hours. Histone H3.3 detected as loading control. **(C)** Titers of PR8 and PR8_CG_ at 48 hpi in human A549 KO cells including parental (black), Vector and LacZ (gray), ZAP KO (green), and KHNYN KO (blue). MOI=0.05. **(D)** Titers of PR8 and PR8_CG_ at 48 hpi in human A549 KO cells including parental (black), Vector (gray), and ZAP+KHNYN KO (green and blue striped). MOI=0.05. *** indicates p≤0.0001 and ** indicates p=0.0045 by one-way ANOVA. **(E)** Titers of PR8 and PR8_CG_ at 48 hpi in human A549 KO cells including parental (black), Vector (gray), and TRIM25 KO (pink). MOI=0.05.

We next tested the ability of endogenous human ZAP and/or KHNYN to restrict CpG-enriched IAV in human lung cells. Therefore, we generated multiple human lung adenocarcinoma A549 clonal cell lines depleted for ZAP, KHNYN, or both by CRISPR/Cas9 genome editing as well as a vector control (Vector) and a negative control guide targeting *E. coli* LacZ beta-galactosidase (LacZ) clonal cell lines (Figure 3A–B). We tested PR8 and PR8_CG_ by multicycle replication assay in these cell lines. We found that single knockout of only ZAP or only KHNYN failed to increase PR8 or PR8_CG_ replication relative to LacZ or parental A549 cells (Figure 3C). We reasoned that this was consistent with our results by overexpression in DF-1 cells such that either ZAP or KHNYN are capable of independently restricting PR8_CG_. Next, we tested multicycle replication of PR8 and PR8_CG_ in double knockout (ZAP and KHNYN depleted) cells revealing that genetic loss of both ZAP and KHNYN increased replication of PR8_CG_ relative to parental A549 or LacZ cell lines (Figure 3D) supporting the hypothesis that ZAP and KHNYN can independently restrict of CpG enriched IAV. We also found replication of PR8 or PR8_CG_ was unaffected by knockout of endogenous TRIM25 (Figure 3E). Altogether, these results support the hypothesis that ZAP and KHNYN can act independently against IAV.

### ROSV is CpG-enriched and susceptible to mammalian CpG-dependent restriction.

**Figure 4:**
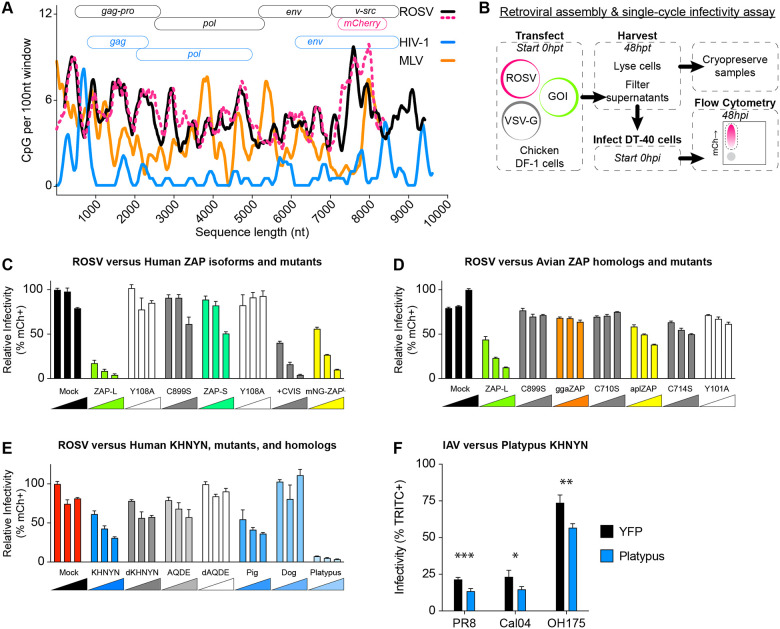
Prenylated human ZAP and mammalian KHNYN independently restrict naturally CpG-enriched ROSV. **(A)** CpG content of retroviruses (ROSV in black, ROSV-mCherry reporter virus in pink dashed line, MLV in orange, and HIV-1 in blue) calculated as # CpG per 100 nucleotides over a sliding window (graph). Schematic of retrovirus open reading frame (ORF) organization (top). **(B)** Schematic of retroviral assembly and single-cycle infectivity assay including plasmid transfection into cells, cell lysate and supernatant harvest at 48 hours post-transfection (hpt), and storage as well as DT40 suspension cell infection and flow cytometry as a measure of infectivity. **(C)** Retroviral assembly and single-cycle infectivity results of human ZAP-L, ZAP-S, and mutants effects on ROSV showing relative percent ROSV infected cells (% mCherry positive; % mCh+) as determined by flow cytometry. DF-1 cells transfected with increasing plasmid amounts of indicated host proteins (100, 200, or 400ng), 1500ng R, and 200ng VSV-G. Infectivity is relative to backbone pcDNA3.2 transfections (Mock). **(D)** Retroviral assembly and single-cycle infectivity results of human ZAP-L, chicken (gga) ZAP, duck (apl) ZAP, and prenylation mutant on ROSV infectivity. **(E)** Retroviral assembly and single-cycle infectivity results of human KHNYN, mutants, and mammalian KHNYN homologues (from pig, dog, and platypus) on ROSV infectivity. **(F)** Single-cycle infectivity of YFP alone (black) and YFP-tagged platypus KHNYN (blue) effects on single-cycle IAV including laboratory PR8, pandemic Cal04, and avian-origin OH175. *** indicates p=0.000832, * indicates p=0.025875, and ** indicates p=0.002263 by unpaired t test.

Avian IAV exhibits an enrichment for CpG content relative to human IAV (Figure 1A, Figures S1–2). We next asked if other avian viruses with different levels of human or mammalian zoonotic interactions might exhibit higher levels of CpG enrichment. We noticed that ROSV (a tractable retrovirus model in chicken) exhibits a higher CpG content relative to mammalian retroviruses, including HIV-1 or MLV. We observed that ROSV is CpG-enriched along its entire length relative to mammalian retroviruses including HIV-1 (Figure 4A). To determine whether ROSV was subject to CpG-dependent restriction, we performed retroviral assembly and single-cycle infectivity assays (schematic in Figure 4B) by co-transfecting chicken DF-1 cells with a full-length ROSV reporter virus expression plasmid and VSV-G along with increasing amounts of control, human ZAP-L, human ZAP-S, KHNYN, or dKHNYN plasmid. We found that human ZAP-L inhibited ROSV, but ZAP-S did not (Figure 4C). Using mutants of human ZAP-L and ZAP-S, we confirmed that for ROSV, ZAP restriction required an intact ZF2 and prenylation motif (Figure 4C). Specifically, the C899S prenylation mutant of ZAP-L failed to restrict ROSV while ZAP-S fused to the four amino acid ZAP-L prenylation motif (+CVIS) gained the ability to restrict ROSV. Next, we tested human ZAP-L, chicken ZAP, and duck ZAP as well as prenylation mutants of each for restriction of ROSV using fusions to mNeongreen. We found that human ZAP-L restricted ROSV but not either of the avian ZAP proteins (Figure 4D). Finally, we tested mammalian orthologues of ZAP from dog, pig, and platypus and found that the prenylated forms of placental mammal ZAP restricted ROSV, while platypus ZAP or a prenylation mutant (C653S) were less restrictive (Figure S7A).

### Mammalian KHNYN restricts ROSV independent of ZAP.

Next, we tested human KHNYN, mutant KHNYN, and mammalian KHNYN orthologues from pig, dog, and platypus for restriction of ROSV. Pigs and dogs are domesticated mammals frequently in contact with agricultural poultry, a common source of avian-to-mammal zoonoses(3). We selected platypus KHNYN to test because it is the only mammalian species to encode KHNYN but lack the NYNRIN gene(45) (Figure S3C). We found that pig KHNYN, but not dog KHNYN, similarly restricted ROSV as human KHNYN while platypus KHNYN potently restricted ROSV (Figure 4E, Figure S7B). We also tested these proteins for ROSV restriction tagged with mNeongreen at their amino terminus relative to catalytically inactivated mutants. Curiously, mutation of the aspartic acid residues in the NYN endonuclease domains inhibited the restrictive ability for pig KHNYN (DD508/508AA) but not platypus KHNYN (DDD416/497/498AAA) (Figure S7B). We tested multiple mutants and truncated forms of platypus KHNYN and found that mutation of aspartic acid residues critical for endonuclease activity in other KHNYN orthologues failed to ameliorate platypus KHNYN activity while truncation mutants which deleted the endonuclease domain lost restriction activity (Figure S7C). We generated structural models of platypus KHNYN and noted a substantial structural difference in the loop above the apex of the putative XG motif in platypus KHNYN encoding a notably shorter loop relative to human, pig, and dog KHNYN (Figure S5E). Curiously, we found that platypus KHNYN also restricted HIV-1, a CpG-enriched HIV-1, and MLV, independent of glycoprotein provided for pseudo-typing (Figure S7D). Finally, we tested if platypus KHNYN could restrict IAV replication in human A549 cell lines expressing YFP-tagged platypus KHNYN. Relative to YFP-alone expressing cells, platypus KHNYN modestly inhibited single cycle PR8, pandemic Cal04, and avian-origin OH175 (Figure 4F). Together, these results support the hypothesis that mammalian ZAP and KHNYN can restrict CpG-enriched avian viruses, including IAV and ROSV. Furthermore, we discovered an orthologue of KHNYN in platypus that potently restricts multiple viruses from distinct virus families independent of CpG content.

## DISCUSSION

KHNYN was identified as a ZAP-interacting cofactor that required ZAP to exert a combined antiviral effect against a CpG-enriched HIV-1(20). Human ZAP functions independent of KHNYN against multiple viruses including HIV-1(19, 37, 49), Ebolavirus(50, 51), Zikavirus(52), and Sindbis virus(27). Here, we provide the first evidence that KHNYN may be capable of exerting an antiviral effect independent of ZAP. We note that, by definition, restriction factors act in a cell-autonomous fashion(53, 54). As such, KHNYN may be a novel *bona fide* restriction factor with unknown RNA preferences Furthermore, multiple groups showed different, sometimes conflicting, results regarding ZAP restriction of IAV(55–58). Here, we observed that human IAV exhibits a marked decrease in CpG content relative to avian IAV (Figure 1 and Figure S1–2). We showed that chicken ZAP as well as duck ZAP do not restrict IAV (Figure 1 and Figure S3–4) or ROSV (Figure 4 and Figure S7). We reasoned that chicken cells (e.g. DF-1) could be used as a gain-of-restriction system to test restriction factors for their ability to inhibit IAV and other CpG-enriched viruses. In DF-1 cells stably expressing human ZAP-L, ZAP-S, KHNYN, a catalytically inactive KHNYN, or TRIM25, we found that ZAP-S and KHNYN modestly restricted PR8 and significantly restricted PR8_CG_ (Figure 2). We also confirmed that this restriction required an intact CpG-recognizing ZF2 of ZAP or a catalytically active KHNYN. We found that human ZAP and KHNYN do not appreciably manipulate the host transcriptome in chicken cells but negatively affect IAV RNA levels (Figure S6). We supported the hypothesis that each of these restriction mechanisms can act independently in human cells wherein only combined knockout of both ZAP and KHNYN in human A549 cells increased replication of PR8_CG_ (Figure 3). We also found that another avian virus is subject to ZAP and KHNYN restriction, ROSV (Figure 4 and Figure S7). Finally, we discovered a potent antiviral KHNYN homologue in platypus capable of restricting multiple divergent retroviruses as well as IAV (Figure 4 and Figure S7). Altogether, we hypothesize that these mammalian restriction factors act against CpG-enriched viruses including avian viruses and select for CpG-depleted nucleotide mutations.

Multiple groups have analyzed the dinucleotide content of influenza sequences, other viruses, as well as host transcripts(11, 13, 16, 18). Here, we analyzed influenza virus segment sequences from different host species collected over the past century and found the CpG content is depleted over time in mammalian species and that this occurs in polymerase segments more so than HA or NP segments (Figure 1). We speculate that over a decades-to-centuries long timeframe, CpG-depletion is a relatively slow adaptative process compared to single amino acid substitutions in PB2, PA, NP, NA, or HA traditionally associated with host species adaptation(59–64). We observed that H1N1 and H3N2 human IAV sequences exhibited a lower CpG content than subtype-matched avian sequences. However, H5N1 and H7N9 sequences were essentially identical in CpG content between human and avian samples likely reflecting the limited amount of time and replication within human patients. Yet, it is unclear how CpG content regulates viral RNA intrinsic stability or viral replication. Recent work showed that human ZAP targets viral RNA molecules encoding a minimum threshold number of CpG dinucleotides within a length of RNA that is neither too dense nor too sparse(65). This likely reflects a requirement for some minimum number of ZAP molecules to bind RNA and if CpG dinucleotides are too close these sites occlude each other while if too sparse or too distributed this dilutes a necessary yet undefined minimum of ZAP clustering. Here, we showed that human ZAP-S can target an artificially CpG-enriched PR8 IAV and to a lesser extent PR8 IAV. Future work should examine how CpG number, density (number per length), segment-specificity, and influenza RNA class (viral messenger, complementary, or genomic RNA) are targeted by ZAP.

Human ZAP-S and ZAP-L can restrict different viruses and human transcripts(66), including those associated with the interferon response(16), based on their differential cellular localization as determined by the carboxy-terminal prenylation motif(22, 37, 58). KHNYN was identified by protein-protein interaction with ZAP in a yeast two-hybrid screen and functionally shown to be a required cofactor for ZAP restriction of an artificially CpG-enriched HIV-1 construct(20). Subsequent work suggested that ZAP-based restriction of different CpG-enriched HIV-1 constructs were not uniformly KHNYN-dependent(49). Curiously, one group showed that ZAP-L requires the C-terminal prenylation motif to restrict CpG-enriched HIV-1(37) while another found that a C-terminally HA-tagged ZAP-L (that effectively eliminates prenylation) restricted different CpG-enriched HIV-1 mutants(19, 44). It is unclear whether the differences in CpG number and density or other technical differences might explain these differences. Previous work demonstrated that human ZAP-L inhibited IAV (including PR8) by binding viral PB2 and PA proteins but is counteracted by PB1(56). However, others found that human ZAP-S inhibits IAV (including PR8) by reducing viral mRNA levels but is counteracted by NS1(57). These two results predated identification of human ZAP preference for CpG dinucleotides. Finally, recent work showed that ZAP-S inhibits PR8 with a CpG-enriched segment 1 but not PR8 with a CpG-enriched segment 5(58). Here, we show that human ZAP-S and KHNYN independently restrict IAV with a CpG-enriched segment 5 (PR8_CG_; Figure 2–3) while ZAP-L and KHNYN independently restrict ROSV (Figures 4). Curiously, the original discovery of ZAP-dependent restriction of MLV identified and used a form of ZAP lacking the carboxy-terminal prenylation motif(67). KHNYN has not been previously shown to independently restrict any viruses. Altogether, we suggest that antiviral activities of ZAP and KHNYN might be context dependent. For example, a subset of viruses might be restricted by the membrane-associated ZAP-L and others restricted by the cytosolic ZAP-S because the antiviral activity of many restriction factors is localization-dependent. In addition, ZAP might restrict some sequences, KHNYN might restrict other sequences, and they may act together against yet a third group of sequences. In fact, the results from Sharp, et al. suggest mutants of a specific virus strain may exhibit differential susceptibility to ZAP and/or KHNYN(58). In the case of segmented viruses, including IAV, this suggests an impact on genetic drift (mutation) as well as genetic shift (reassortment) over time.

In contrast to ZAP, relatively less is known about the function, preferences, and regulation of KHNYN. KHNYN is one member of family of paralogous genes encoding a putative RNA-binding domain fused to a NYN-endonuclease domain(46). Our phylogenetic analysis (Figure S5) support the hypothesis that KHNYN may be derived from N4BP1 occurring prior to the radiation of mammalian species and NYNRIN is the result of a gene duplication and fusion/integration of KHNYN to Metaviral *Pol* gene occurring between the emergence of monotreme and therian mammals(45). We showed here that mutation of key aspartic acid residues in the NYN domain of KHNYN reduce its restriction ability, similar to others(20). We also show that mutations in the KH-domain of KHNYN reduces its restriction ability(47). Recent structural and biochemical studies showed the extended di-KH domain of KHNYN does not bind RNA but the full-length KHNYN protein binds RNA without CpG preferences and the NYN domain exhibits single-stranded endonuclease specificity(68). Furthermore, how these preferences and activity change over evolutionary time in unclear. Finally, while we identified serendipitously the potent platypus KHNYN restriction factor, its mechanism of action is unclear. We speculate that this potent ancestral KHNYN required attenuation for the genesis of retroelement-derived NYNRIN and retroviral-associated placentation acquired during the rapid evolutionary leap from monotremes to therian mammals between 180–160 million years ago(45, 69).

## LIMITATIONS

We appreciate limitations exist in this study attempting to synthesize and understand the multiple (and sometimes conflicting) reports regarding CpG-depletion in cytoplasmic-resident RNAs and viral adaptation driven by ZAP, KHNYN, and related antiviral genes. Throughout this study we primarily use a common laboratory strain of IAV and a previously published CpG-enriched segment 5 mutant strain (PR8 and PR8_CG_). Future work will benefit from examining ZAP, KHNYN, and TRIM25 susceptibility of many influenza strains derived from wild birds, agricultural poultry, and domestic mammals as well as emerging mammalian H5N1 IAV isolates. This work largely uses chicken cell lines and relies on overexpression of many host restriction factors by transfection or stable transduction. While aspects of our work were substantiated by knockdown and/or knockout of endogenous host cell factors, future work will benefit from using primary cells. Furthermore, future work will also benefit from cell systems derived from avian reservoir species including shorebirds that currently do not exist as tractable laboratory models. Finally, we acknowledge two aspects of KHNYN antiviral activity shown here are limited. Specifically, the mechanisms of KHNYN antiviral activity should be fully elucidated in the future including potential co-factors. In addition, the potent antiviral platypus KHNYN orthologue is derived from a predicted mRNA and protein product and investigation of its mechanism of action will be vastly informative. However, the sum of the results shown here should spur multiple important research directions and warrant intense interest in multiple fields including host-pathogen interactions, virus evolution, mammalian evolution, and embryonic development.

## METHODS

### Cell lines.

Mammalian cells, chicken DF-1 cells, and duck CCL-141 cells were grown in DMEM supplemented with 10% fetal bovine serum (FBS) and 1% penicillin/streptomycin. Chicken DT40 cells were grown in DMEM supplemented with 10% FBS, 10% tryptose phosphate buffer, 1% penicillin/streptomycin, and 50μM beta-mercaptomethanol. Cells were maintained at 37°C, 5% CO_2_, and 50% humidity. HEK293T (male human epithelial kidney, ATCC, CRL-3216), A549 (male human lung carcinoma, ATCC, CCL-185), PK-15 (male porcine kidney, ATCC, CCL-33), MDCK (female canine kidney, ATCC, CCL-34), DF-1 (unspecified sex chicken embryonic fibroblast, ATCC, CRL-12203), DT40 (unspecified sex chicken lymphoblast, ATCC, CRL-2111), and CCL-141 (unspecified sex duck embryonic fibroblast, ATCC) cells were obtained from ATCC and used without extensive passaging. Sup-T11 (male human T lymphoblast clone derived from Sup-T1, ATCC, CRL-1942) cells have been described previously(70).

### Plasmids.

A CpG-enriched IAV segment 5 (encoding nucleoprotein, NP) derived from Gaunt, et al.(43), ordered from IDT as a gBlock, and cloned into a pDZ bidirectional influenza vRNA/mRNA expression plasmid(71) via In-Fusion (Takara Bio). Expression plasmids for human ZAP-L, ZAP-S, KHNYN, and TRIM25 as well as pig ZAP, chicken ZAP, and pig KHNYN were generated using conventional molecular biology techniques. Specifically, RNA was extracted from human A549 cells, chicken DF-1, or pig PK15 cells using Qiagen RNeasy Mini Kit, cDNA synthesized using QIAGEN Quantitect cDNA Synthesis Kit, and genes of interest were amplified by PCR from first-strand cDNA, restriction enzyme digested, and ligated into bespoke MLV-based retroviral vectors also harboring blasticidin genes downstream of an IRES for bicistronic expression(72–74) or bespoke pcDNA3-based expression vectors. Dog and platypus KHNYN as well as dog and platypus ZAP were ordered from IDT as gBlocks. Duck ZAP was synthesized by Twist Biosciences followed by subcloning into additional plasmid backbones. Mutants, chimeras, and fluorescently tagged versions of all plasmids were generated by site-directed mutagenesis and/or overlapping PCR and subcloned into expression vectors using Phusion DNA polymerase and T4 DNA ligase (NEB). RCAS-GFP plasmid was a gift of Connie Cepko (Addgene #13878). ROSV-mCherry was generated by replacing EGFP in RCAS-GFP with mCherry followed by site-directed mutagenesis to inactivate the Env glycoprotein open reading reframe. HIV-mCherry has been described previously(75). HIV_CG_-mCh was cloned by inserting a gBlock (IDT) encoding env86–561CpG(20) (from NCBI: MN685350.1) into an HIV-1 NL4–3 Env- Vpr- Nef- mCherry+ (HIV-mCh) reporter virus expression plasmid. MLV-Gag-mCh was a kind gift of Nathan Sherer(76). Codon-optimized HIV-1 NL4–3 Env (SynEnv) was synthesized as a gBlock by IDT prior to subcloning into pcDNA3.2. pCAG-RABV-G (Addgene #36398) expressing Rabies virus glycoprotein (RABV-G) was a kind gift of Connie Cepko(77). CRISPR/Cas9 editing of human A549 cells was performed by lentiviral transduction using pLentiCRISPR1000(78). Attempts at genetic editing in avian cells with pLentiCRISPR1000 were unsuccessful. CRISPR/Cas9 editing of avian cells was performed by sequential retroviral transduction of Cas9-GFP expression vector followed by single guide RNA (sgRNA) expression vector – both bespoke plasmids generated for this study. Briefly, guide RNAs were designed from the literature, Synthego design tool, or RGEN Cas-Designer(79), complementary oligos with Esp3I restriction enzyme compatible ends were annealed, and subsequently ligated into pLentiCRISPR1000 or pMLV.miRFP670.iU6sgRNA via Golden-Gate ligation with T4 DNA ligase. IAV bidirectional rescue plasmids (pDZ) have been described previously(80). Lentiviral constructs for shRNA knockdown of chicken ZAP were cloned by annealing complementary oligos with AgeI and EcoRI restriction enzyme compatible ends and subsequently ligated into pLKO.2 via Golden-Gate ligation with T4 DNA ligase. shRNAs were designed targeting chicken ZC3HAV1 using the Broad Institute’s Genetic Perturbation Platform. DNA constructs were sequence confirmed by whole plasmid sequencing performed by Plasmidsaurus using Oxford Nanopore Technology or Sanger sequencing performed by Eurofins Scientific or GeneWiz from Azenta. Plasmid, shRNA, siRNA, and sgRNA sequences are provided in Supplemental File 1.

### Retroviral transductions and stable cell generation.

Simple retroviruses for transduction were generated by transfecting HEK293T cells with 1μg vector plasmid(73, 74), 1μg pMD-gagpol(81), and 200ng pMD-VSVG(82) using Transit-LT1 (Mirus Bio), changing media after 24 hours, and harvesting culture supernatant at 48 hours post-transfection followed by 0.45μm syringe filtration. Lentiviruses for transduction were generated by transfecting HEK293T with 1.25μg pLentiCRISPR1000 or pLKO.2_shRNA plasmid, 750ng psPAX2 (Addgene plasmid #12260; encoding Gag/GagPol, Rev, Tat), and 200ng pMD-VSVG, changing media after 24 hours, and harvesting culture supernatant at 48 hours post-transfection followed by 0.45μm syringe filtration. Transducing virus preps were aliquoted and stored at −20°C. Stable cells were generated as previously described(75, 83). Briefly, approximately 2500 target cells were seeded into a 96-well flat bottom plate, allowed to adhere overnight, and 20–200μL of transducing viral supernatant with up to 10μg/mL polybrene added to each well. After 2–3 days in culture, transduced cells are washed with PBS, and fresh media containing antibiotic (2μg/mL puromycin, 2μg/mL blasticidin, or 200μg/mL hygromycin; GoldBio, Inc) was added. Cells were grown and expanded in the ongoing presence of antibiotic. In the case of CRISPR/Cas9 lentiviral transduction, A549 cells were single cell cloned by serial dilution (re-plating from pooled transducing culture into 96-well flat bottom plates at 25, 5, 1, and 0.2 cells per well or by gradient dilution down and across 96-well plates). After 2–4 days, single cell-derived colonies were marked and followed regularly, expanded, and then screened initially by western blot or PCR. Multiple independent clones were screened for each knockout. Genetic knockout in A549 cells was confirmed by western blot following genomic DNA extraction, amplification of Cas9 target site, blunt cloning into pJET1.2 (Thermo Scientific), and Sanger sequencing multiple transformed clones. Double knockout cells were transduced simultaneously (A549.ZK1 and A549.ZK2) with ZAP-targeting (blasticidin-resistant) and KHNYN-targeting (puromycin-resistant) CRISPR/Cas9 lentiviruses, selected with both antibiotics after 48 hours, single cell cloned, screened by western blot or PCR, and confirmed by Sanger sequencing. Avian cells were transduced with simple retrovirus Cas9-GFP (puromycin-resistant). Avian cell knockouts were maintained as pools stably expressing Cas9 and sgRNAs (blasticidin-resistant). Genetic knockout in avian cells was determined by genomic DNA extraction, PCR amplification of Cas9 target site, Sanger sequencing of pooled amplicons, and computational deconvolution of overlapping chromatograms by TIDE analysis(84).

### Influenza viruses.

Wild-type Influenza A/Puerto Rico/8/1934(H1N1) (PR8) was rescued by plasmid-based transfection into HEK293T cells using Lipofectamine 3000 (ThermoFisher Scientific) and amplified in 10-day old embryonated chicken eggs. PR8_CG_ virus was made by plasmid-based transfection with the WT segment 5 plasmid replaced with CpG-enriched segment 5 and amplified in embryonated chicken eggs. Single-cycle PR8 and Cal04 (A/California/04/2009(H1N1)) lack virus-encoded HA and instead express mCherry from the HA segment were rescued by plasmid-based transfection into HEK293T cells and amplified in MDCK cells stably expressing PR8 HA(85). Chimeric OH175 Influenza A/Green-Winged Teal/Ohio/175/1986(H2N1) viruses(61) were rescued by plasmid-based transfection into HEK293T cells using six internal segment plasmids from OH175, the HA, and NA segment plasmids from PR8 for biosafety reasons and subsequently amplified in MDCK cells. PR8, PR8_CG_, and OH175 transfections were performed in combination with pCAGGS expression plasmids as needed. OH175 plasmids (also known as S009) were a kind gift of Andrew Mehle. Viral titers were determined by plaque assay in MDCK cells as previously described(86).

### Influenza multicycle replication assays.

For multicycle IAV infections, 500,000 A549 or DF-1 cells were plated in 6-well plates (or 100,000 CCL-141 in 12-well) and allowed to grow overnight for up to 24 hours. Cells were infected at an MOI of 0.05 infecting a confluent culture of cells, as previously described(86). For each cell condition (e.g. DF-1 Vector, ZAP, KHNYN) and for each virus (e.g. PR8 or PR8_CG_) there were three independently infected well replicates. Briefly, cells were washed with PBS prior to addition of 1mL infection media (1x PBS, 2.5% bovine serum albumin, 1% calcium/magnesium). Viral stocks were diluted such that 40μL of inoculum could be added to each well yielding an MOI of 0.05 plaque forming units per cell (pfu/cell). After addition of viral inoculum, cells were incubated at 37°C for 1 hour. Next, cells were washed with PBS prior to addition of 2.5mL viral growth medium (1x DMEM, 2.5% HEPES buffer, 2.5% bovine serum fraction V [7.5%], 1% penicillin/streptomycin) supplemented with TPCK-resistant trypsin (1μg/mL for A549 as well as 0.625 μg/mL for DF-1 and CCL-141). Culture supernatants (at least two aliquots of 200μL each) were collected immediately after addition to wells (0 hours post-infection) and at 24- and 48-hours post-infection (hpi) and optionally at 12-hours post-infection. Supernatants were stored at −80°C until viral titer was determined by plaque assay in MDCK cells as previously described(86). Multicycle replication assays were performed in triplicate with key results being replicated in independent assays (e.g. ZAP-S relative to Vector in Figure 2B, 2C, and 2D). Plaque assays were performed and quantified single-blinded. For transient knockdown by siRNA, 100,000 CCL-141 cells were plate in 12-well plates and transfected with mock, 5nM scramble control, and 1nM or 10nM pools of 10 duplex siRNAs targeting *Anas platyrhynchus* ZC3HAV1 (aplZAP) using RNAiMAX (ThermoFisher). siRNAs were designed using IDT dsiRNA design tool and sequences are provided in Supplemental File 1.

### Single-cycle retrovirus assembly and infectivity assay.

For ROSV, 500,000 DF-1 cells were plated into 6-well plates in 2mL media, cultured overnight, and transfected with 1.6μg RCAS-mCherry, 200ng pMD-VSVG, 100/200/400ng gene of interest plasmid, and topped up to 2.2μg total plasmid DNA mass with pBlueScript using TransIT-LT1 (Mirus Bio). At 24 hours post-transfection, we exchanged media. At 48 hours post-transfection, virus-containing supernatants were harvested and filtered using 3mL syringes and 0.45μm syringe PVDF filters. Transfections were performed as dose-titrations of gene of interest plasmid amounts rather than triplicates of a single plasmid amount. Culture supernatants were used to infect chicken DT40 suspension cells in triplicate and the remaining volume was stored at −80°C. We performed flow cytometry to detect infected (i.e. mCherry-positive) DT40 cells at 48 hours post-infection using a Becton Dickinson FACS Canto II with a high-throughput plate-based sample aspirator. For MLV, HEK293T cells were plated and transfected with 500ng pMD-gagpol, 1100ng pMLV-Gag-mCherry, 200ng pMD-VSVG, 100/200/400ng gene of interest plasmid, and topped up to 2.2μg total plasmid DNA mass with pBlueScript. For HIV-1, HEK293T cells were plated and transfected with 1600ng pHIV-mCherry, 200ng glycoprotein-expression plasmid (pMD2-VSVG, pCAG-RABVG, or pcDNA.HIV.SynEnv), 100/200/400ng gene of interest plasmid, and topped up to 2.2μg total plasmid DNA mass with pBlueScript. MLV single-cycle infectivity was detected by infecting HEK239T cells and HIV-1 infectivity was detected using SupT11 cells (for VSV-G and HIV Env pseudo-typed virus) or HEK293T cells (for RABV-G pseudo-typed virus).

### Western blotting and antibodies.

A549 cells were treated with 1000U/mL of universal interferon-alpha (PBL Assay Sciences, #11200–2) for 24 hours prior to pelleting cells and lysing in 2.5X reducing sample buffer (RSB; 125 mM Tris HCl, 20% glycerol, 7.5% SDS, 5% β-mercaptoethanol, 250 mM DTT, 0.05% Orange G, pH 6.8). Western blots were performed conventionally using commercial precast polyacrylamide gels (26-well Bio-Rad, 4–20% Criterion TGX, #5671095) for electrophoresis (60 volts for 60 minutes followed by 120 volts for 60 minutes) and transferred to 0.2μm nitrocellulose membrane using Invitrogen Power Blotter XL (ThermoFisher Scientific; ≤25 volts for 12 minutes). Membranes were washed in PBS and PBST (PBS with 0.1% Tween-20) followed by blocking (2% dry milk in PBST). With constant rocking at all steps, membranes were incubated with primary antibodies overnight at 4°C, followed by three washes for five minutes each with PBST, incubation with secondary antibodies in 2% milk in PBST for at least one hour at room temperature, followed by three washes for five minutes each in PBST, and one final wash in PBS. Primary antibodies used include rabbit anti-ZAP (Abcam, ab154680, 1:5,000 dilution), rabbit anti-KHNYN (Invitrogen, PA5–31660, 1:500), rabbit anti-TRIM25 (Abcam, ab167154, 1:10,000), and rabbit anti-H3 (Abcam, ab1791, 1:10,000). Secondary antibodies used include goat anti-mouse IgG IRDye 680LT and goat anti-rabbit IgG IRDye 800CW (both used at 1:10,000 dilution in 2% milk in PBST). Membranes were imaged using a LiCor Odyssey Fc infrared fluorescent imager for 10 minutes per channel.

### Fluorescence microscopy.

Fixed-cell images were obtained on a BioTek Cytation 5 (Agilent) using a 4X (NA=0.13, #1220519) or 20X (NA=0.40, #1220517) Plan Fluorite objective and LED-filter cube sets for DAPI (excitation = 377±50nm; emission = 447±60nm; #1225007 and #1225100), GFP for mNeonGreen (excitation = 469±35; emission = 525±39; #1225101 and #1225001), and TRITC for mCherry and AlexaFluor568 (excitation = 556±29; emission = 600±37; #1225125 and #1225012). We quantified single-cycle IAV infections by imaging and used the BioTek Gen5 software to calculate percent infectivity using scPR8 (MOI=5) and scCal04 (MOI=5) encoded mCherry as a readout for infectivity (# TRITC+ cells / # DAPI-stained nuclei). To quantify OH175 non-replicating infections, we performed immunofluorescence on infected cells (MOI=0.5 without TPCK trypsin to block virus transmission) fixed with 4% paraformaldehyde at 24 hours post-infection. Briefly, we subjected cells to permeabilization (PBS with 0.25% Triton X-100) for 10 minutes, blocking (PBS with 3% bovine serum albumin), primary antibody staining with mouse anti-NP (BEI, NR-43899, 1:500) in blocking buffer (3% bovine serum albumin) for one hour, secondary antibody staining with goat anti-rabbit AlexaFlour-568 (Invitrogen, #A-11031, 1:500), and nuclear counterstaining with DAPI (Sigma, D9542, 100ng/mL).

### Structural models of ZAP and KHNYN.

For structural models of ZAP, amino acid sequences for the four zinc finger domains of different species and mutants of ZAP were used as input for RobettaCM using the human ZAP crystal structure (PDB: 6UEJ) for comparative modeling. We noted that in most cases, *ab initio* folding by AlphaFold or RoseTTAFold may not capture metal ion-coordinated finger structures. Sequences used are provided in Supplemental File 1. Models generated and 6UEJ are shown in Supplemental File 4. For structural models of KHNYN, amino acid sequences for the KH domain of KHNYN were used as input for RoseTTAFold using default settings as well as RobettaCM using HNRNPK KH-domain (PDB: 1ZZK) for comparative modeling. Sequences used are shown in Supplemental File 1. Both RoseTTAFold and RobettaCM are publicly available at robetta.bakerlab.org. All models generated, 1ZZK, and 6Q3V (N4BP1 KH-domain) are provided in Supplemental File 5 and 6. Models were visualized in Chimera (v1.13.1, build 41965, UCSC) and aligned using MatchMaker with a Needleman-Wunsch alignment algorithm and BLOSUM-62 matrix.

### Phylogenetic analyses.

Coding sequences (CDS) of human KHNYN, NYNRIN, N4BP1, and their orthologues were downloaded from ensembl. CDS of ZAP homologues were downloaded from ensemble. CDS were aligned using ClustalOmega in Seaview (V5.0.4). Phylogenetic trees were generated in Seaview using PhyML with 100 bootstraps using CDS. Synteny schematics were generated manually in Adobe Illustrator using data from UCSC ENCODE Genome Browser, NCBI, and ensembl. Alignments and phylogenetic trees are available in Supplementary Data 7.

### Virus bioinformatic analysis.

Full-length influenza virus sequences were downloaded from the Influenza Research Database for each animal host species (human A, avian A, swine A, human B, and human C) including the nucleotide sequence, host, collection date, and subtype. Dinucleotide values were counted by string count. Data were plotted using ggplot2. Data and R code are available in Supplemental File 3. Sliding window CpG data were analyzed using an in-house custom script (cpg_counts.R) available in Supplemental File 3.

### RNA-seq.

RNA was extracted from mock and infected samples at 24 hours post-infection (MOI=0.05) using Qiagen RNeasy Kit. Samples were submitted for polyA-selected RNA (mRNA) sequencing using an Illumina NovaSeq 6000 with 150 bp paired end reads at the University of Illinois Urbana-Champaign Roy J. Carver Biotechnology Center. Reads were trimmed of adapter sequences and low-quality reads eliminated using Trimmomatic (V0.32). Trimmed reads were mapped to a hybrid reference genome (Gallus gallus GRCg7b, PR8, and PR8_CG_) using STAR (V2.7.11b) and quantified per feature using HTSeq (V2.0). Differential expression analysis was performed using DESeq2 (v1.34.0). Data were processed and analyzed in R-Studio version 1.3.1073 using R language version 3.6.3. RNA-seq data are available at the NCBI Sequence Read Archive (SRA) under accession code XXX.

### Quantitative reverse-transcription PCR.

To quantify endogenous chicken *ZC3HAV1* and *TUBA4A* mRNA transcripts in shRNA-expressing stable cells following chicken interferon-alpha treatment (1000U/mL; Bio-Rad) for 24 hours, 500,000 cells were plated in 6-well plates and allowed to adhere overnight in culture media followed by addition of chicken interferon or vehicle. After 24 hours, cells were harvested, RNA extracted using Qiagen RNeasy kit, and cDNA synthesized used Qiagen Quantitect Reverse Transcription Kit using random hexamers and poly-dT primers with genomic DNA wipeout. Quantitative PCR was performed in technical triplicate on a LightCycler 480 II (Roche Life Science) using Ssofast Eva Green Supermix (Bio-Rad).

### Statistical analyses.

Plaque assay results from IAV infections were plotted in GraphPad Prism 10 and one-way ANOVA was used at 48 hours post-infection or two-way ANOVA for multiple time points to determine statistical significance. Single-cycle ROSV and IAV infectivity results were plotted in GraphPad Prism 10. Unpaired t tests were used to calculate statistical significance for IAV single-cycle infectivity assays. Statistical analyses are available in Supplemental File 2.

## Supplementary Material

Supplement 1

Supplement 2

Supplement 3

Supplement 4

Supplement 5

Supplement 6

Supplement 7

1

## SIGNIFICANCE

Viruses adapt to hosts to replicate successfully. We show that two mammalian proteins, ZAP and KHNYN, restrict CpG-enriched avian viruses. Mammalian KHNYN may be a *bona fide* restriction factor with cell-autonomous activity. We also identified a platypus KHNYN with potent and broad antiviral activity highlighting a significant need to investigate antiviral mechanisms in novel and understudied species. Ongoing efforts to understand viruses with zoonotic potential will benefit from further identification of species-/class-specific restriction factors and their antiviral preferences. Furthermore, we speculate that evolving viral nucleotide composition indicates zoonotic potential and adaptation to mammals requires dinucleotide and amino acid changes.

## HIGHLIGHTS:

- CpG content is depleted in human and swine IAV relative to avian IAV
- Human ZAP-S and KHNYN but not chicken ZAP independently restrict CpG-rich IAV.
- Mammalian ZAP-L and KHNYN but not chicken ZAP independently restrict ROSV.
- Platypus KHNYN potently restricts retroviruses, including IAV, HIV-1, MLV, and ROSV.
