# Supplementary figures and images for: Mammalian ZAP and KHNYN can independently restrict CpG-enriched avian viruses
Source: bioRxiv. 2025 Sep 10:2024.12.23.629495. Originally published 2024 Dec 23. Preprint. [Version 2] doi: 10.1101/2024.12.23.629495 (PMC11703154; doi:10.1101/2024.12.23.629495)

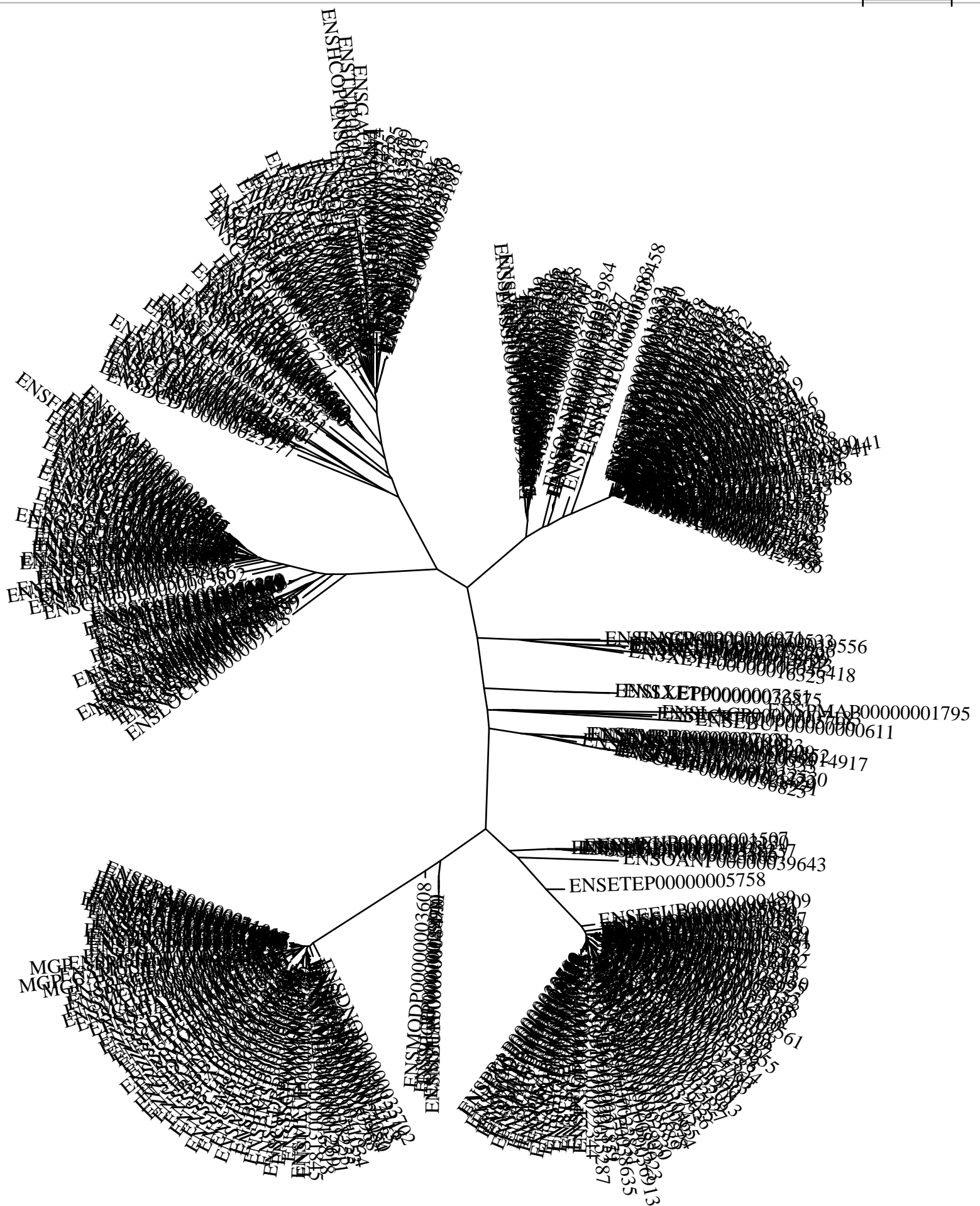

Supplement: Supplement 8 [file media-8.zip › FigS5C_PhyML_circular.pdf]

PhyML ln(L)=-145363.9 4623 sites GTR 100 replic. 4 rate classes

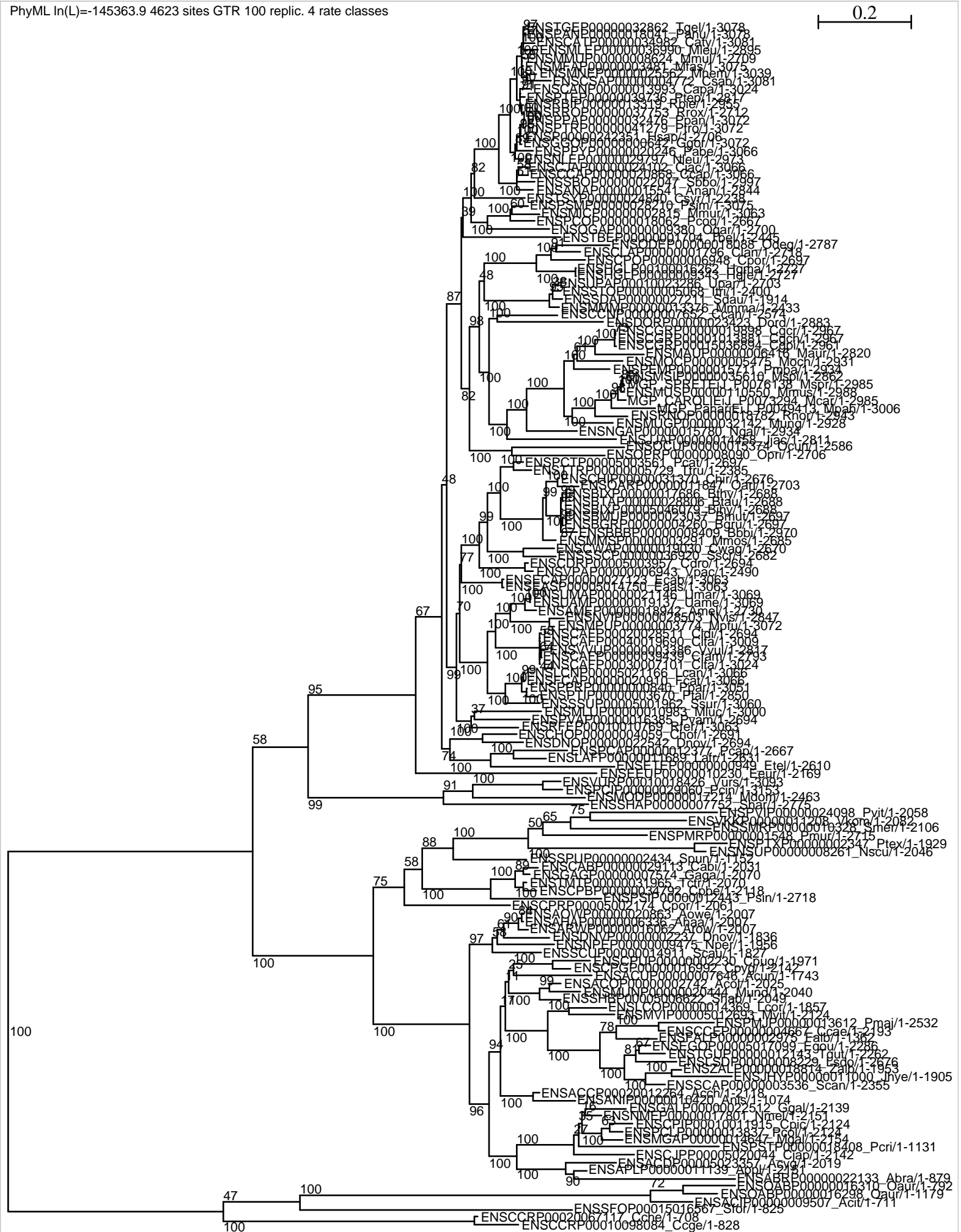

Supplement: Supplement 9 [file media-9.zip › Combined_ZC3HAV1_orthologues-PhyML_tree.pdf]
