# Supplementary material for: Mammalian ZAP and KHNYN can independently restrict CpG-enriched avian viruses
Source: bioRxiv. 2025 Sep 10:2024.12.23.629495. Originally published 2024 Dec 23. Preprint. [Version 2] doi: 10.1101/2024.12.23.629495 (PMC11703154; doi:10.1101/2024.12.23.629495)

bioRxiv preprint doi: https://doi.org/10.1101/2024.12.23.629495; this version posted September 10, 2025. The copyright holder for this preprint (which was not certified by peer review) is the author/funder, who has granted bioRxiv a license to display the preprint in perpetuity. It is made available under aCC-BY 4.0 International license.

SUPPLEMENTAL FIGURE LEGENDS

S1 Fig. Mammalian IAV sequences are CpG-depleted over time relative to avian IAV.
(A) IAV sequences from human (blue) and swine (green) hosts exhibit depletion of CpG content (CpG per kb = # CpG / segment length in kilobases (kb)) relative to avian (orange) IAV over time since 1918. IBV (pink) and ICV (purple) exhibit relatively stable but dramatically lower CpG content than all IAV segments. Thick lines represent linear trend of data surrounded by 95% confidence interval in lighter shading.
(B) CpG content of IAV sequences from avian, human, and swine segregated by virus segment. Segments 1-3 include PB2, PB1, and PA encoding the viral Polymerase. Segments 4-6 include HA, NP, and NA encoding virion proteins often subject to adaptive immune pressure. Segments 7-8 include M and NS.
(C) CpG content of IAV sequences from avian, human, and swine hosts segregated by common HA and NA subtypes.
(D) CpG content of IAV sequences segregated by time periods indicated on the x-axis (top). Schematic of IAV natural history showing avian H1N1, H2N1, and H3N2 in orange shaded lines, human circulating subtypes in shaded blue lines, 2009 H1N1 pandemic swine flu in green lines, (middle) and source of reassortant segments in matched colors (bottom).
Thick black bars represent median CpG content, boxes define the 25th to 75th percentile, and whiskers represent range excluding outliers. Horizontal dashed gray lines highlight median avian IAV CpG content as a reference.

S2 Fig. Dinucleotide content of influenza viruses.
(A) CpG content as the normalized rho (ρ = #CpG / length divided by the product of #C / length and #G per length) of all IAV sequences from avian, human, and swine hosts as well as IBV and ICV.
(B) Percent GC content (%GC = (#G + #C) / total length) all IAV sequences from avian, human, and swine hosts as well as IBV and ICV.
(C) GpC content of all IAV sequences from avian, human, and swine hosts as well as IBV and ICV.
(D) Remaining dinucleotide content of all IAV sequences from avian, human, and swine hosts as well as IBV and ICV.

bioRxiv preprint doi: https://doi.org/10.1101/2024.12.23.629495; this version posted September 10, 2025. The copyright holder for this preprint (which was not certified by peer review) is the author/funder, who has granted bioRxiv a license to display the preprint in perpetuity. It is made available under aCC-BY 4.0 International license.

S3 Fig. Structural comparison of avian ZAP proteins and data for avian ZAP expression and knockdowns.

(A) Structure and model of human (blue; PDB: 6UEJ), chicken (RobettaCM, orange), chicken (RoseTTAFold, medium orange), and chicken (AlphaFold2, light orange) ZAP zinc fingers (1-4) with a CpGpU RNA oligonucleotide (gray; PDB: 6UEJ).

Structure and model of human (blue; PDB: 6UEJ), duck (RobettaCM, green), duck (RoseTTAFold, medium green), and duck (AlphaFold2, light green) ZAP zinc fingers (1-4) with a CpGpU RNA oligonucleotide (gray; PDB: 6UEJ).

Structure and model of human (blue; PDB: 6UEJ), quail (RobettaCM, pink), quail (RoseTTAFold, medium pink), and quail (AlphaFold2, light pink) ZAP zinc fingers (1-4) with a CpGpU RNA oligonucleotide (gray; PDB: 6UEJ).

Structure and model of human (blue; PDB: 6UEJ), chicken (RobettaCM, orange), duck (RobettaCM, green), and chicken (AlphaFold2, light pink) ZAP zinc fingers (1-4) with a CpGpU RNA oligonucleotide (gray; PDB: 6UEJ).

(B) Root mean squared deviation (RMSD) of structures and models in (A-D).

(C) Western blots of chicken DF-1 cells stably expressing mNG, mNG-ggaZAP, and untagged ggaZAP as well as Vector transduced cells detecting mNG and blasticidin S deaminase (BSD). Actin detected as a loading control.

(D) Western blots of chicken DF-1 cells stably expressing mNG, mNG-ggaZAP, and mutants of mNG-ggaZAP as well as Vector transduced cells detecting mNG and BSD. Actin detected as a loading control.

(E) qPCR results detecting endogenous gga *ZC3HAV1* mRNA in chicken DF-1 cells transduced with lentiviruses expressing shRNAs indicated (shades of gray) following treatment with 1000 U/mL of chicken interferon-alpha (IFN) relative to mock treated cells normalized to gga *TUBA1A* using a 2^{-ddCt} calculation.

(F) Titers of PR8 and $PR8_{CG}$ over 48 hpi in chicken DF-1 cells stably expressing shRNAs targeting endogenous ggaZAP (shggaZAP1-3, shades of gray triangles/diamonds/hexagons), a control scrambled sequence siRNA (shScramble, dark gray squares), and parental untransduced DF-1 cells (black circles). MOI=0.05.

(G) Titers of PR8 and $PR8_{CG}$ at 48 hpi in duck CCL-141 cells transiently transfected with 1nM or 10nM siRNAs targeting endogenous aplZAP (siZAP, shades of yellow), a control scrambled sequence siRNA (siNeg, 5nM, gray), and parental untransfected (black) CCL-141 cells. MOI=0.05.

bioRxiv preprint doi: https://doi.org/10.1101/2024.12.23.629495; this version posted September 10, 2025. The copyright holder for this preprint (which was not certified by peer review) is the author/funder, who has granted bioRxiv a license to display the preprint in perpetuity. It is made available under aCC-BY 4.0 International license.

(H) Western blots of chicken DF-1 cells stably expressing human ZAP-L, ZAP-S, KHNYN, and TRIM25 as well as dually transduced ZAP-L or ZAP-S with KHNYN or dKHNYN including Vector transduced cells detecting ZAP and KHNYN. GAPDH detected as a loading control.

S4 Fig. TIDE analysis of pooled CRISPR/Cas9 chicken DF-1 and duck CCL-141 ZC3HAV1/ZAP knockouts.

(A-B) Schematic depicting exon organization of chicken ZC3HAV1/ZAP (orange) and duck ZC3HAV1/ZAP (yellow) as well as sites targeted by CRISPR single guide RNAs (sgRNAs; scissors). Colored boxes indicate coding exons and white boxes indicate noncoding portions of exons. Genomic sequence targeted by sgRNAs (underlined) and protospacer adjacent motif (PAM, lowercase) and amino acids encoding by that region (bold residues overlap sgRNA target). Genomic distances are relative and total locus size indicated.

(C) Total knockout efficiency as determined by tracking of indels by decomposition (TIDE) analysis (% Knockout) in each pool relative to LacZ control cells.

(D-F) TIDE analysis showing percent of sequences in pool exhibiting indicated indel (-10 to +10 bp from expected cut site) in chicken ZC3HAV1-targeted KO DF-1 cells.

(G-I) TIDE analysis showing percent of sequences in pool exhibiting indicated indel (-10 to +10 bp from expected cut site) in duck ZC3HAV1-targeted KO CCL-141 cells.

Red bars indicate disruptive indels (CRISPR-mediated insertions or deletions resulting in out-of-frame mutations). Gray bars indicate un-edited sequences. Black bars indicate in-frame indels (CRISPR-mediated insertions or deletions resulting in amino acid deletions).

S5 Fig. Genetic and genomic comparisons of KHNYN genes

(A) Synteny diagrams of KHNYN genomic organization in representative species. KHNYN gene in blue, NYNRIN in magenta, neighboring human/pig/koala genes in black, neighboring platypus genes in red, and human/chicken genes syntenic to red genes in gray. Distances are relative and approximate size of locus shown on the right in kilobasepairs (Kbp).

(B) Schematic of exon organization (left) and protein domain architecture (right) of human N4BP1 (pink), KHNYN (blue), and NYNRIN (magenta). KH domain represents the extended HNRNPK-like, GXXG-like motif residues typical of KH-domains are indicated, NYN represents the NYN/PIN endonuclease domain, and CU represents cullin-binding domain associated with NEDD8 (KHNYN and NYNRIN) or cousin of CUBAN domain (N4BP1).

(C) Maximum likelihood phylogeny of all ensembl-annotated orthologues of human KHNYN, NYNRIN, and N4BP1.

bioRxiv preprint doi: https://doi.org/10.1101/2024.12.23.629495; this version posted September 10, 2025. The copyright holder for this preprint (which was not certified by peer review) is the author/funder, who has granted bioRxiv a license to display the preprint in perpetuity. It is made available under aCC-BY 4.0 International license.

(D) Synteny diagrams and schematics of exon organization for alligator, anole, and zebrafish *"khnyn-like"* genes.

(E) Structural predictions in three views of human (dark blue), pig (medium blue), dog (cyan), and platypus (lime green) KHNYN (residues 1-213). Putative GXXG motif highlighted by dashed black circle. Platypus-specific short loop highlighted by dashed black square.

S6 Fig. YFP-tagged human ZAP-S and KHNYN inhibit PR8$_{CG}$ and OH175 IAV.

(A) Titers of PR8, PR8$_{CG}$. and avian OH175 at 48 hpi in chicken DF-1 cells stably expressing YFP-tagged human ZAP-L (orange), human ZAP-S (green), chicken ggaZAP (orange/gray), KHNYN (blue), dKHNYN (gray) and platypus oanKHNYN (pale blue) relative to YFP alone (black). MOI=0.05.

(B) Western blots of chicken DF-1 cells used in (A) detecting YFP. Histone H3 detected as a loading control.

(C) CpG content of OH175 (shades of red by segment length), OH175 segment 3 PA bolded line, as well as PR8 segment 3 PA (bold blue line) calculated as # CpG per 100 nucleotides (nt) over a sliding window (top). Schematic of PR8 and OH175 viruses and displaying CpG content for each virus and total CpG number encoded by each PA segment (bottom).

(D) Fold enrichment by qPCR for PR8 IAV segment 5 NP RNA following GFP-Trap immunoprecipitation of indicated YFP-tagged proteins from chicken DF-1 cells uninfected or infected with PR8 or PR8$_{CG}$. MOI=5. Fold enrichment is shown relative to parental chicken DF-1 cells lacking YFP, similar to an IgG isotype control used in chromatin immunoprecipitation (ChIP-qPCR) using a 2^{-ddCt} calculation. Western blots detecting YFP in IP (top) and YFP and IAV NP in Input (bottom). Actin detected as loading control.

S7 Fig. Chicken and IAV transcriptome largely unaffected by human ZAP or KHNYN.

(A) Principal component analysis (PCA) of RNA-seq data.

(B) Heatmap showing differentially expressed IAV mRNAs.

(C) Heatmap showing most differentially expressed chicken mRNAs.

S8 Fig. Platypus KHNYN is a potent and broadly acting antiviral protein.

(A) Retroviral assembly and single-cycle infectivity results of mammalian ZAP-L and ZAP-S homologues including pig, dog, and platypus on ROSV infectivity (relative infectivity, %mCh+). Platypus encodes a well annotated ZAP-L homologue but not a ZAP-S, a C653S prenylation mutant was included.

bioRxiv preprint doi: https://doi.org/10.1101/2024.12.23.629495; this version posted September 10, 2025. The copyright holder for this preprint (which was not certified by peer review) is the author/funder, who has granted bioRxiv a license to display the preprint in perpetuity. It is made available under aCC-BY 4.0 International license.

(B) Retroviral assembly and single-cycle infectivity results of mammalian KHNYN homologues including pig, dog, and platypus as well as catalytically inactivated (*"d"*) mutants of each on ROSV infectivity.

(C) Retroviral assembly and single-cycle infectivity results of platypus KHNYN including truncation mutants on ROSV infectivity (top). Schematic of protein domain architecture of platypus KHNYN with arrows indicating position of truncating stop mutation (bottom).

(D) Retroviral assembly and single-cycle infectivity results of platypus KHNYN including truncation mutants on ROSV infectivity (top). Indication (bottom) of virus tested (HIV-mCh, HIV_{CG}-mCh, or MLV-mCh) and glycoprotein used for pseudotyping (HIV-1 Env, VSV-G, or RABV-G).

(E) Retroviral assembly and single-cycle infectivity results of mammalian KHNYN homologues including pig, dog, and platypus as well as catalytically inactivated (*"d"*) mutants of each on ROSV infectivity in chicken ZAP knockout DF-1 cells (ggaZAP-3 KO from **Fig 1G, S4C Fig, and S4F Fig**).

S9 Fig. ZAP localization conferred by prenylation motif in mammal and avian species.

(A) Fluorescence images of DF-1 cells expressing mNG as well as fusion proteins of mNG-ZAP orthologues and mutants indicated (green). Cell and nuclei outline in dashed white lines. Scale bars represent 10 microns.

(B) Amino acid alignment of CaaX prenylation motif from multiple species indicated.

S10 Fig. Conservation of mammalian KHNYN endonuclease domain and mammalian KHNYN subcellular localization.

(A) Amino acid alignment of NYN/PIN endonuclease domain (human residues 437-589) from multiple species KHNYN orthologues indicated.

(B) Structural predictions in three views of human (dark blue), pig (medium blue), dog (cyan), and platypus (lime green) KHNYN (residues 437-589). Catalytic aspartic acid residues highlighted by dashed black circle.

(C) Fluorescence images of DF-1 cells expressing mNG as well as fusion proteins of mNG-KHNYN orthologues and mutants indicated (green). Cell and nuclei outline in dashed white lines. Scale bars represent 10 microns.

S1 Table. Key reagents. Spreadsheet

S2 Table. Statistical analyses and graphed values. Spreadsheet

bioRxiv preprint doi: https://doi.org/10.1101/2024.12.23.629495; this version posted September 10, 2025. The copyright holder for this preprint (which was not certified by peer review) is the author/funder, who has granted bioRxiv a license to display the preprint in perpetuity. It is made available under aCC-BY 4.0 International license.

S1 File. Influenza sequences analysis R code. Text files

S2 File. DEseq2 analysis R code. Text files

S1 Data. Human and avian ZAP structural models. Python files open in UCSC Chimera

S2 Data. Mammalian KHNYN structural models of KH region. Python files open in UCSC Chimera

S3 Data. Mammalian KHNYN structural models of NYN domain. Python files open in UCSC Chimera

S4 Data. N4BP1, KHNYN, and NYNRIN phylogenetic data. Clustal nucleotide alignment, pdf of phylogenetic tree

S5 Data. ZC3HAV1/ZAP phylogenetic data. Clustal nucleotide alignment, pdf of phylogenetic tree

S1 Fig

bioRxiv preprint doi: https://doi.org/10.1101/2024.12.23.629495; this version posted September 10, 2025. The copyright holder for this preprint (which was not certified by peer review) is the author/funder, who has granted bioRxiv a license to display the preprint in perpetuity. It is made available under aCC-BY 4.0 International license.

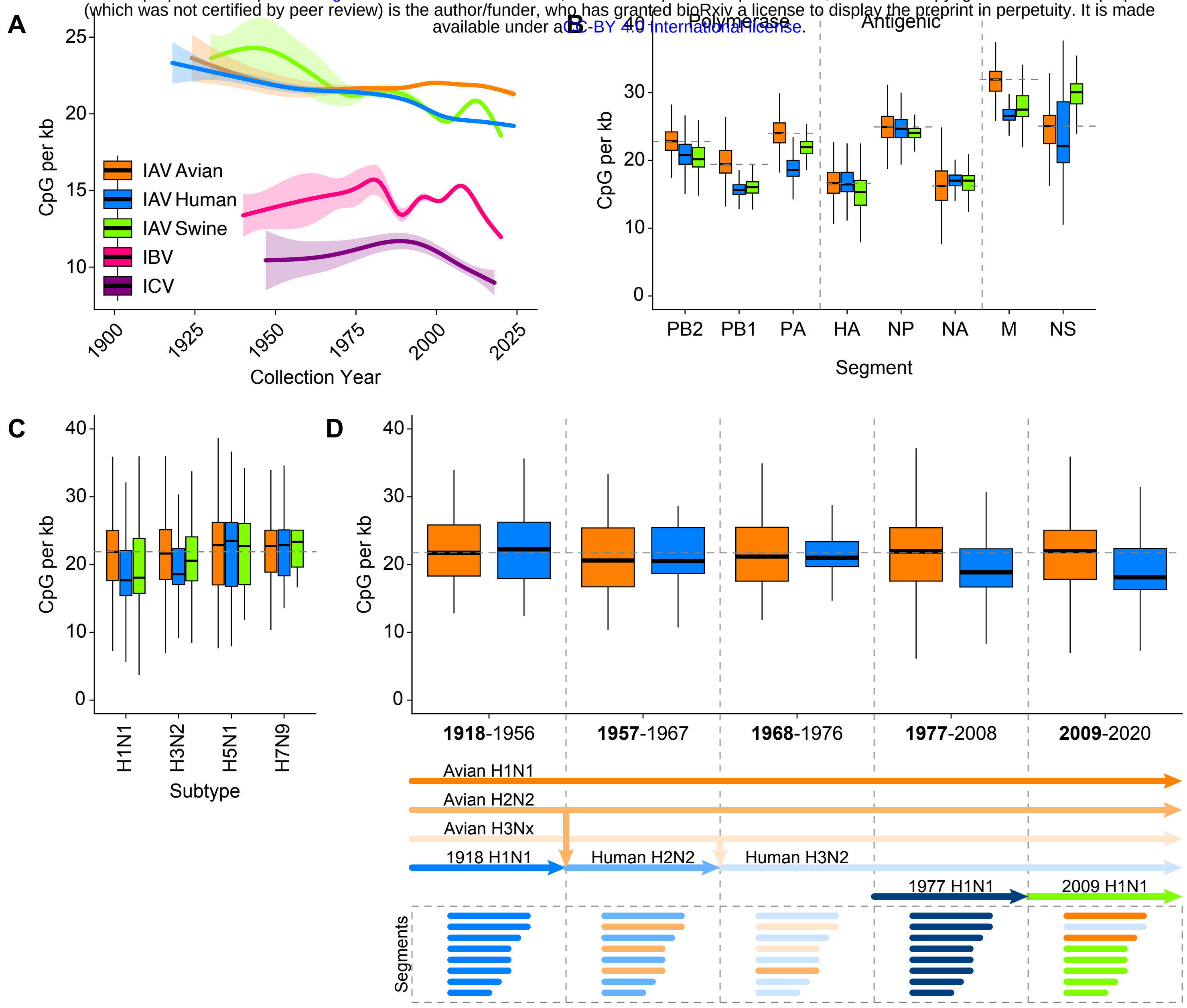

S2 Fig

bioRxiv preprint doi: https://doi.org/10.1101/2024.12.23.629495; this version posted September 10, 2025. The copyright holder for this preprint (which was not certified by peer review) is the author/funder, who has granted bioRxiv a license to display the preprint in perpetuity. It is made available under aCC-BY 4.0 International license.

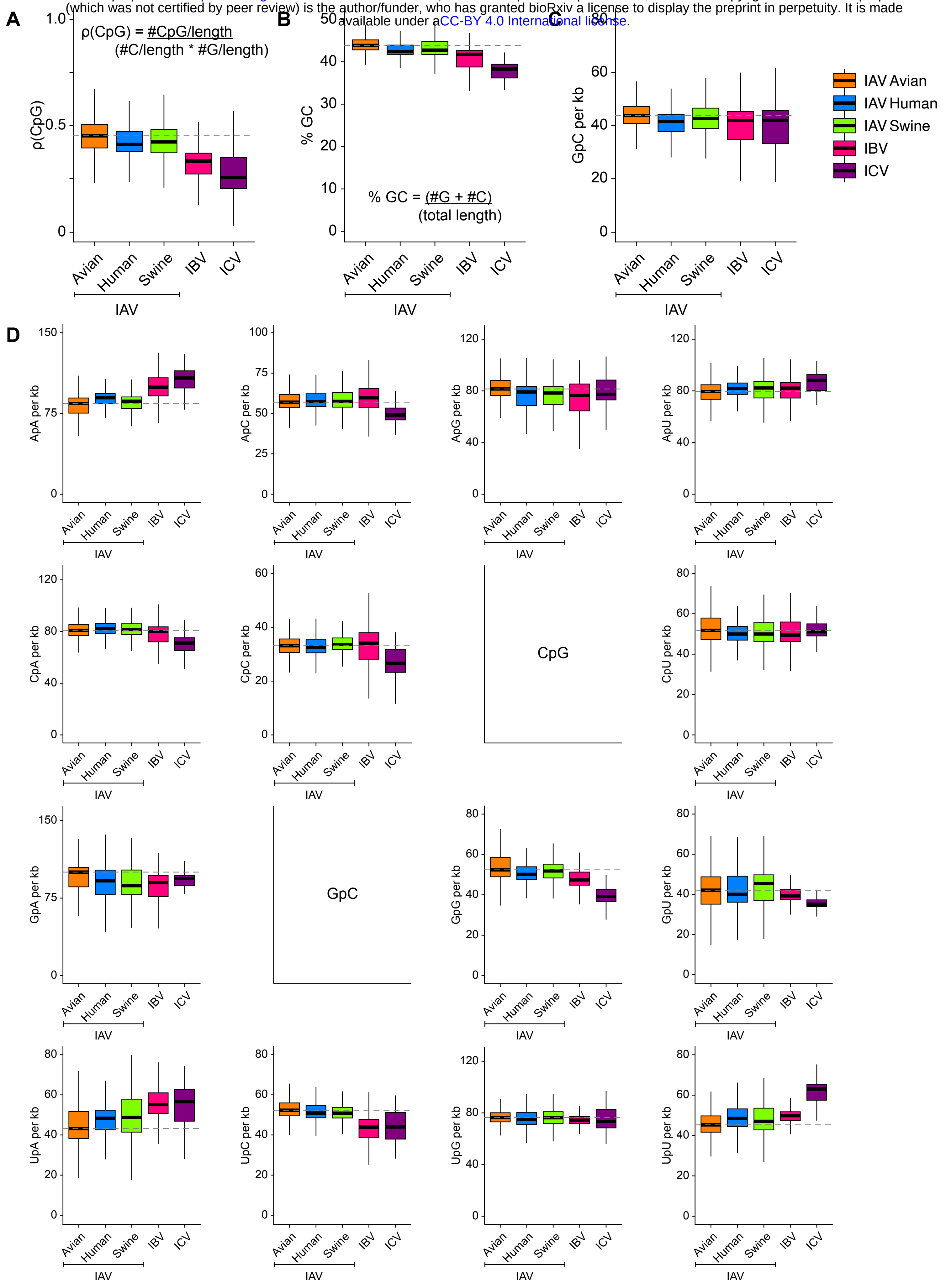

S3 Fig

bioRxiv preprint doi: https://doi.org/10.1101/2024.12.23.629495; this version posted September 10, 2025. The copyright holder for this preprint (which was not certified by peer review) is the author/funder, who has granted bioRxiv a license to display the preprint in perpetuity. It is made available under aCC-BY 4.0 International license.

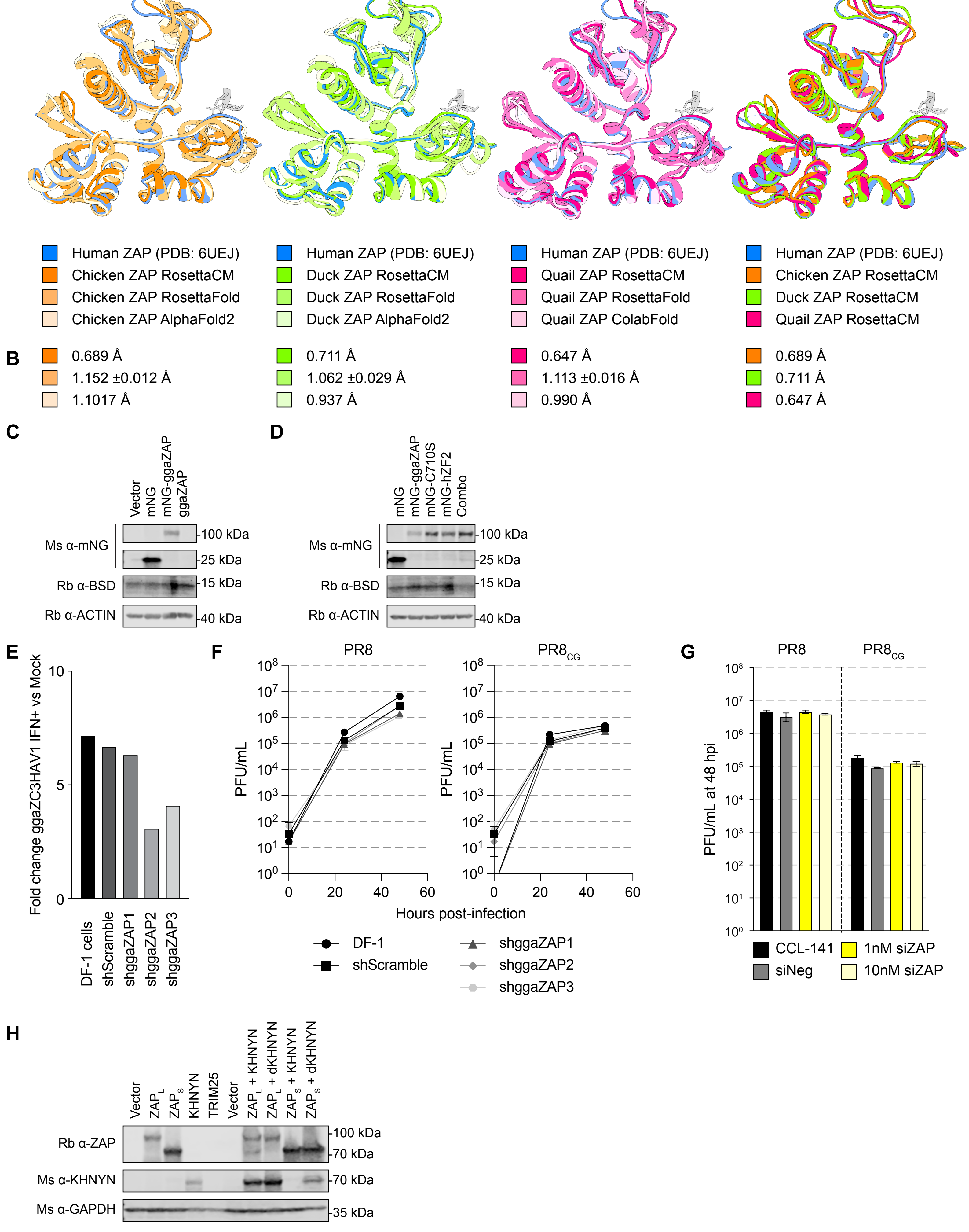

S4 Fig

bioRxiv preprint doi: https://doi.org/10.1101/2024.12.23.629495; this version posted September 10, 2025. The copyright holder for this preprint (which was not certified by peer review) is the author/funder, who has granted bioRxiv a license to display the preprint in perpetuity. It is made available under aCC-BY 4.0 International license.

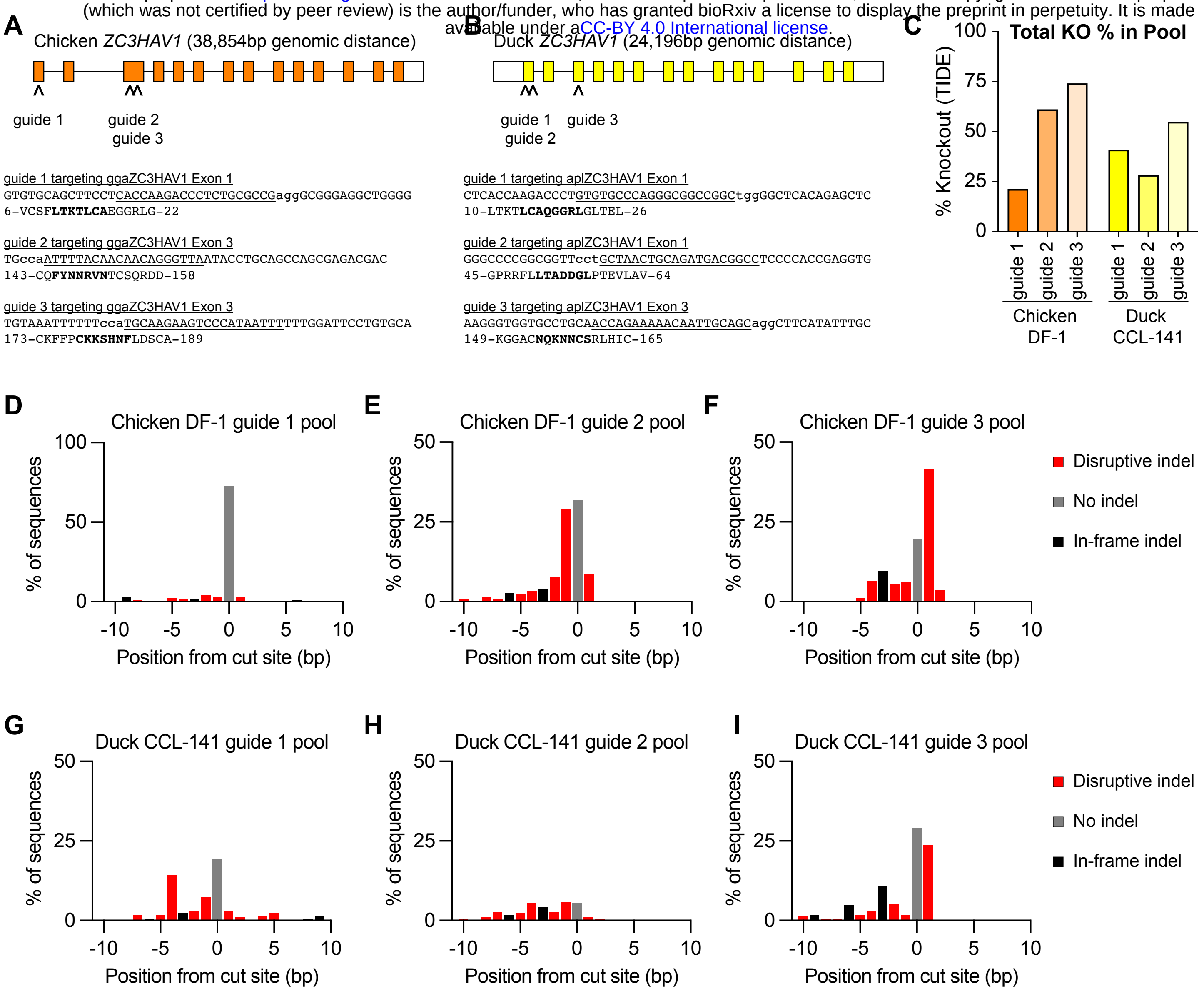

S5 Fig

bioRxiv preprint doi: https://doi.org/10.1101/2024.12.23.629495; this version posted September 10, 2025. The copyright holder for this preprint (which was not certified by peer review) is the author/funder, who has granted bioRxiv a license to display the preprint in perpetuity. It is made available under aCC-BY 4.0 International license.

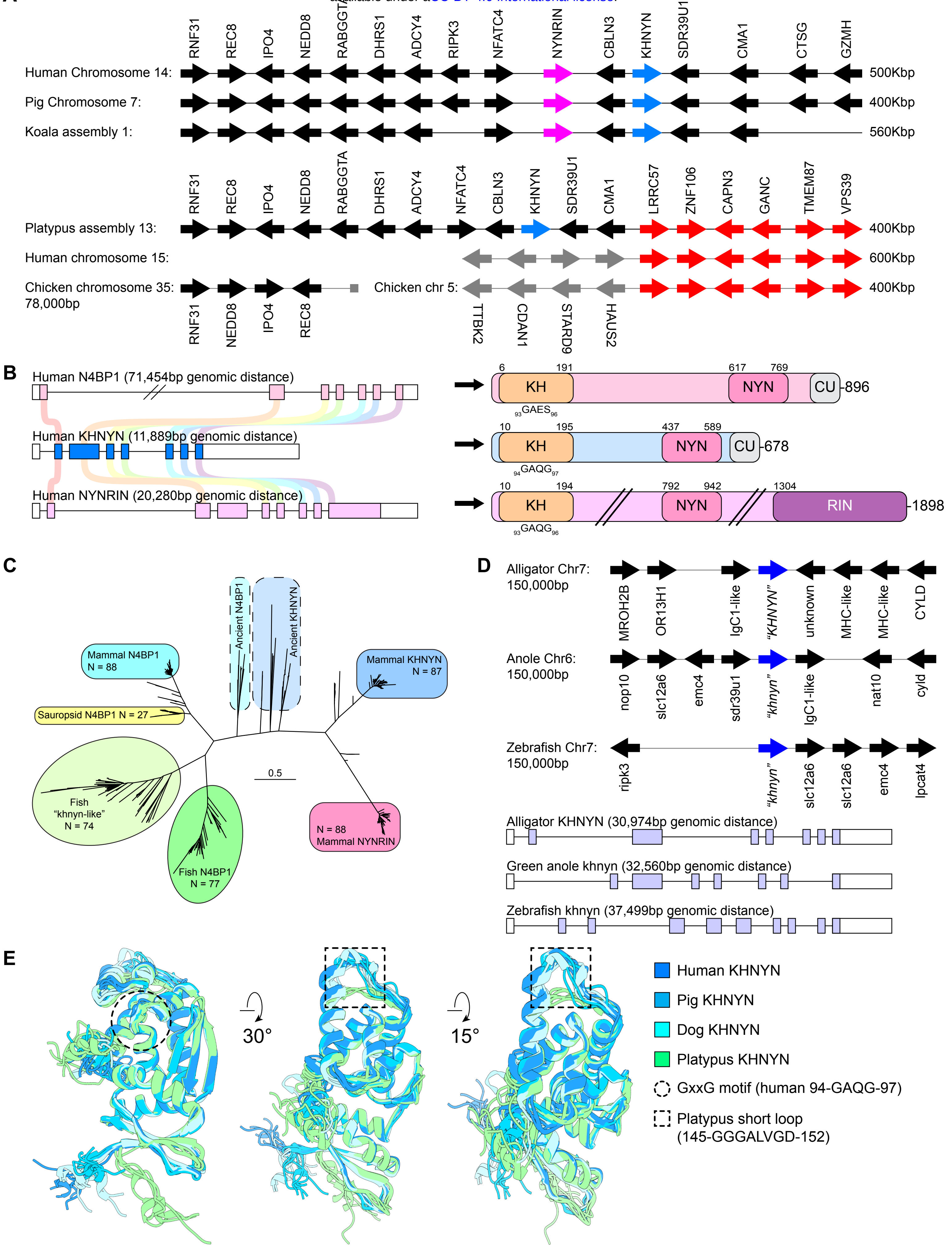

S6 Fig

bioRxiv preprint doi: https://doi.org/10.1101/2024.12.23.629495; this version posted September 10, 2025. The copyright holder for this preprint (which was not certified by peer review) is the author/funder, who has granted bioRxiv a license to display the preprint in perpetuity. It is made available under aCC-BY 4.0 International license.

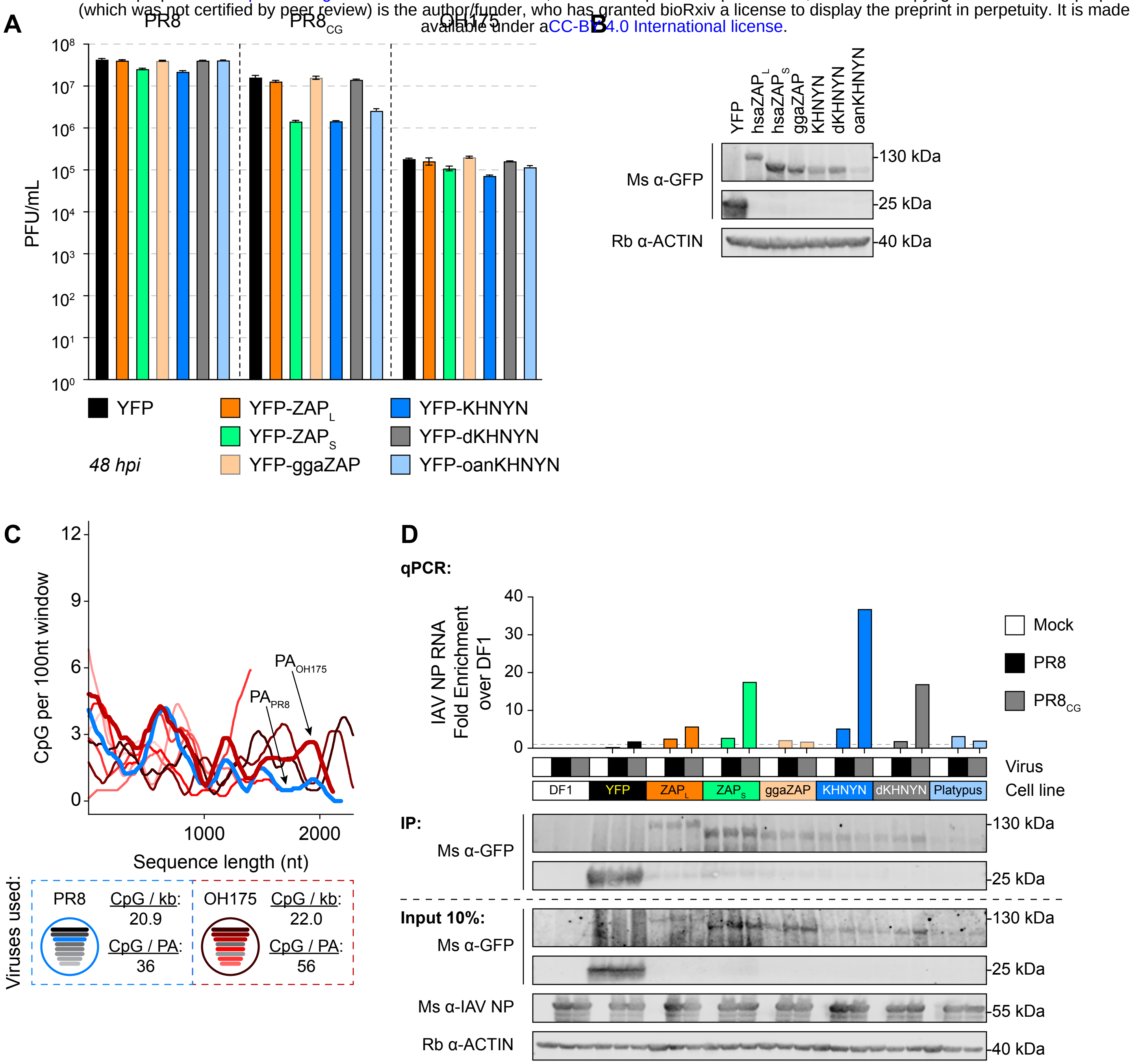

S7 Fig

bioRxiv preprint doi: https://doi.org/10.1101/2024.12.23.629495; this version posted September 10, 2025. The copyright holder for this preprint (which was not certified by peer review) is the author/funder, who has granted bioRxiv a license to display the preprint in perpetuity. It is made available under aCC-BY 4.0 International license.

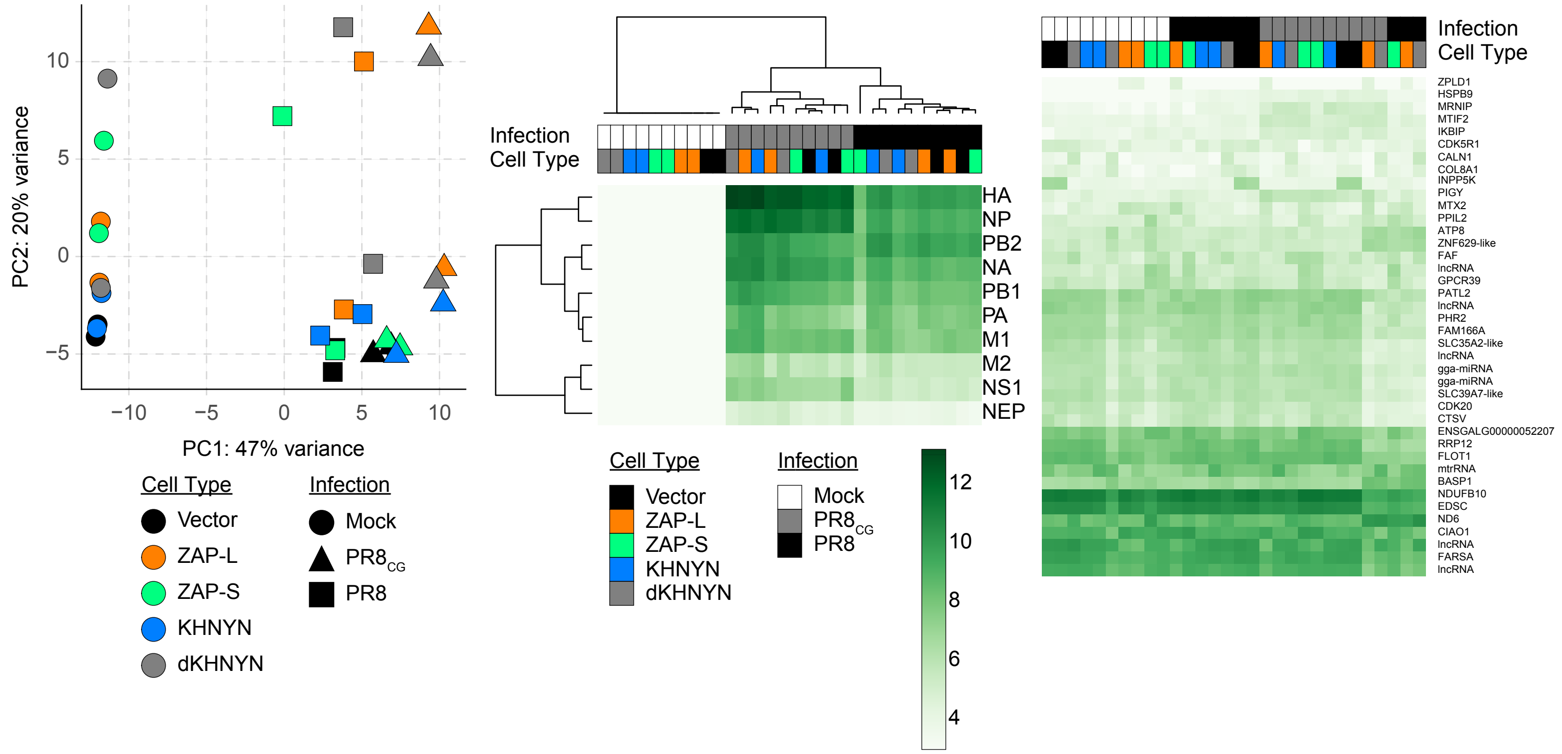

S8 Fig

bioRxiv preprint doi: https://doi.org/10.1101/2024.12.23.629495; this version posted September 10, 2025. The copyright holder for this preprint (which was not certified by peer review) is the author/funder, who has granted bioRxiv a license to display the preprint in perpetuity. It is made available under aCC-BY 4.0 International license.

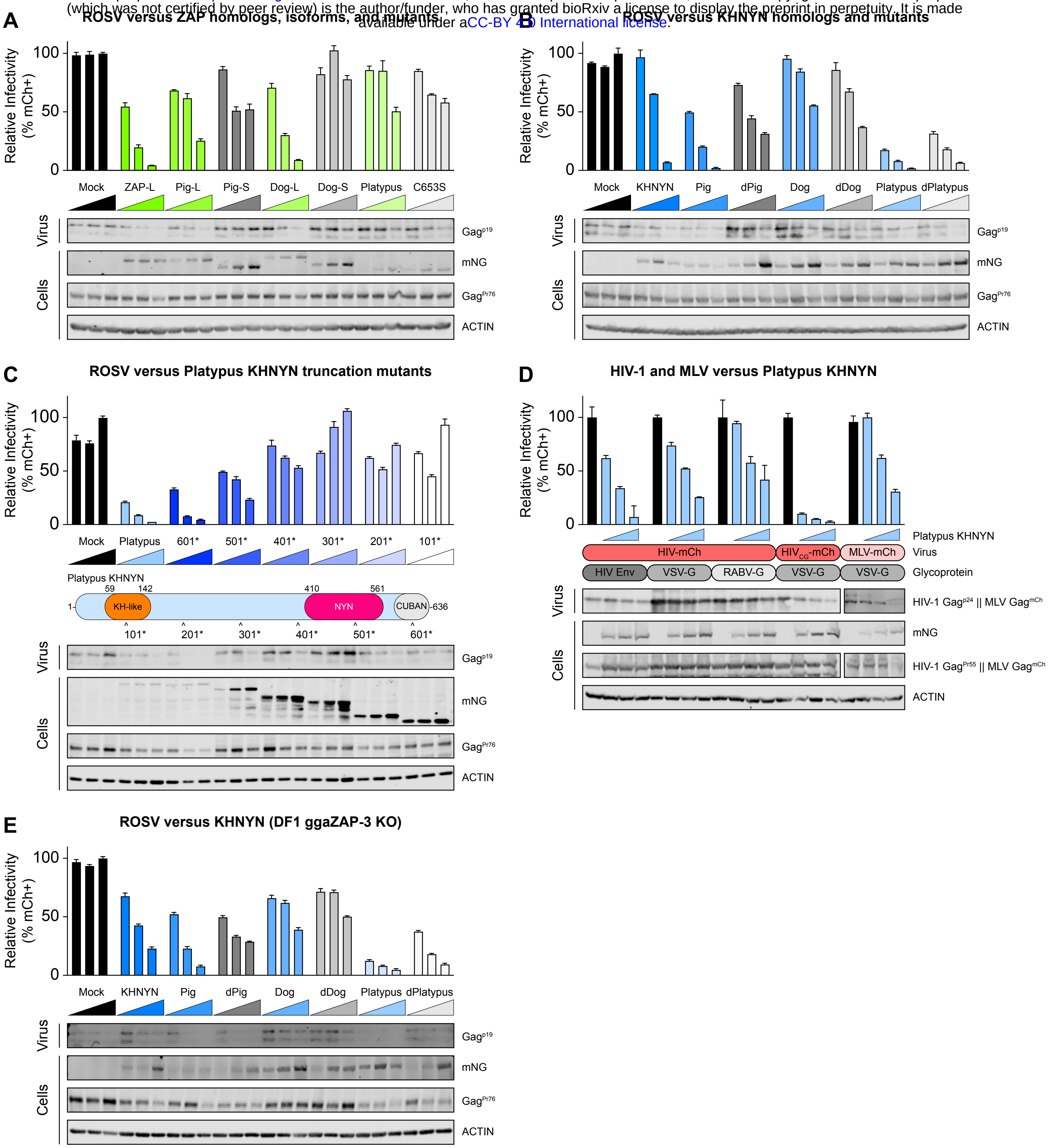

S9 Fig

bioRxiv preprint doi: https://doi.org/10.1101/2024.12.23.629495; this version posted September 10, 2025. The copyright holder for this preprint (which was not certified by peer review) is the author/funder, who has granted bioRxiv a license to display the preprint in perpetuity. It is made available under aCC-BY 4.0 International license.

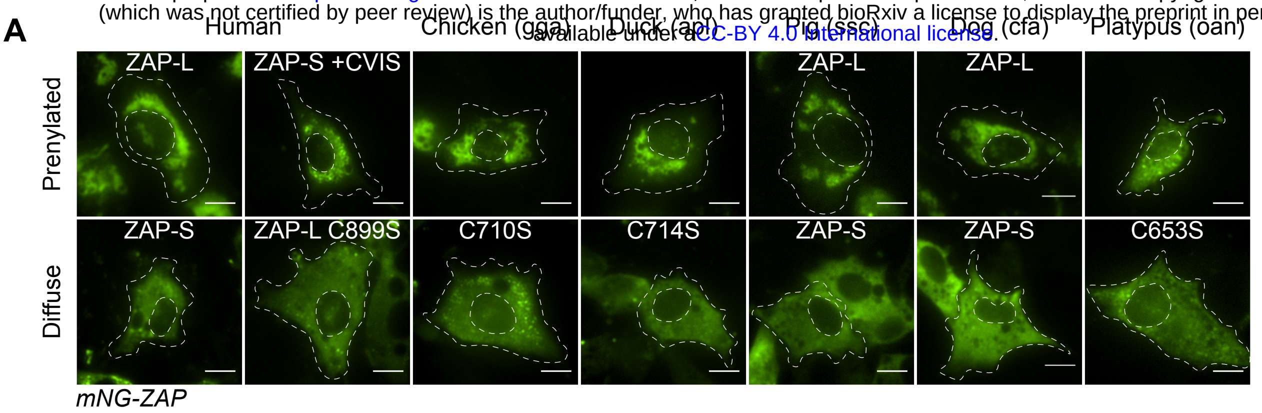

B

**Human**	CVIS*
Chimpanzee	....
**Pig**	....
**Dog**	....
Cat	....
Horse	....
Mouse	.I..
Rat	.I..
**Platypus**	.NLQ
**Chicken**	.IVC
Quail	.IV.
**Duck**	.I..

bioRxiv preprint doi: https://doi.org/10.1101/2024.12.23.629495; this version posted September 10, 2025. The copyright holder for this preprint (which was not certified by peer review) is the author/funder, who has granted bioRxiv a license to display the preprint in perpetuity. It is made available under aCC-BY 4.0 International license.

S10 Fig

A

```
           437                                                                         513
Human      LRHIVIDGSNVAMVHGLQHYFSSRGIAIAVQYFWDRGHRDITVFVPQWRFSKDAKVRESHFLQKLYSLSLLSLTPSR
Chimpanzee .............................................................................
Pig        .....................................................S....G...H..............
Dog        ..........................................................G..................
Cat        ..........................................................G..................
Horse      ...................H.................................S....G.Y................
Mouse      ...........................L.........................S.......................
Rat        ...V.......................L.........................S.......................
Platypus   ...VI............H.V..C..V.L...........E.........LK..P..K.G...TI.QD.R...F....

           514                                                                        589
Human      VMDGKRISSYDDRFMVKLAEETDGIIVSNDQFRDLAEESEKWMAIIRERLLPFTFVGNLFMVPDDPLGRNGPTLDE
Chimpanzee ............................................................................
Pig        .L..........................................................................
Dog        ........................V...................................................
Cat        ........................V...................................................
Horse      .....................................................................H..M...
Mouse      .......................................D....................................
Rat        .......................................D....................................
Platypus   .V.....TP......L........V..T...LQ...K..GE.FE..S...........I..........K......
```

B

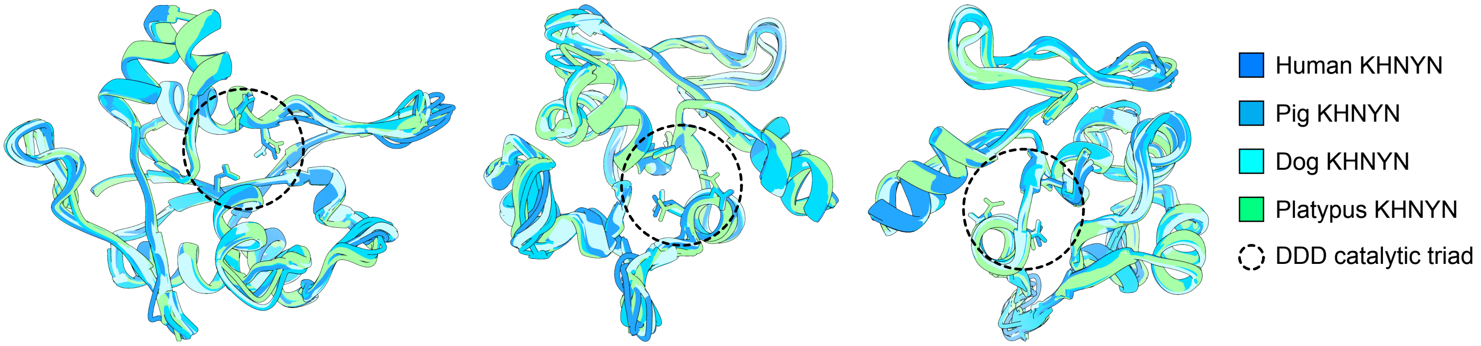

C

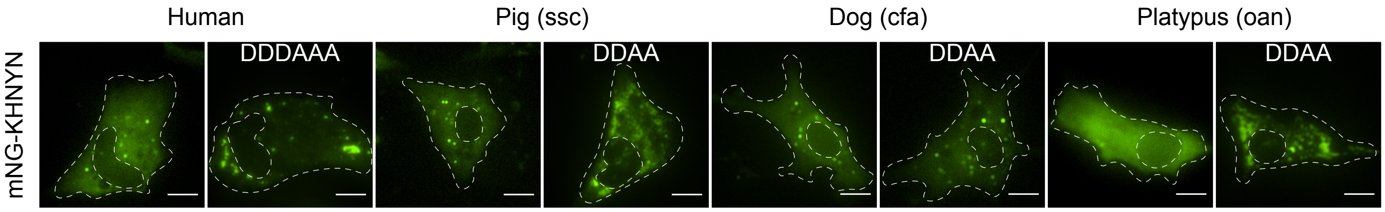

Supplement: Supplement 10 [file NIHPP2024.12.23.629495v2-supplement-10.pdf]
