# Supplementary material for: Mammalian ZAP and KHNYN independently restrict CpG-enriched avian viruses
Source: bioRxiv. 2024 Dec 23:2024.12.23.629495. Preprint. [Version 1] doi: 10.1101/2024.12.23.629495 (PMC11703154; doi:10.1101/2024.12.23.629495)

bioRxiv preprint doi: https://doi.org/10.1101/2024.12.23.629495; this version posted December 23, 2024. The copyright holder for this preprint (which was not certified by peer review) is the author/funder, who has granted bioRxiv a license to display the preprint in perpetuity. It is made available under aCC-BY 4.0 International license.

SUPPLEMENTAL FIGURE LEGENDS

Figure S1 Mammalian IAV sequences are CpG-depleted over time relative to avian IAV.
(A) IAV sequences from human (blue) and swine (green) hosts exhibit depletion of CpG content (CpG per kb = # CpG / segment length in kilobases (kb)) relative to avian (orange) IAV over time since 1918. IBV (pink) and ICV (purple) exhibit relatively stable but dramatically lower CpG content than all IAV segments. Thick lines represent linear trend of data surrounded by 95% confidence interval in lighter shading.
(B) CpG content of IAV sequences from avian, human, and swine segregated by virus segment. Segments 1-3 include PB2, PB1, and PA encoding the viral Polymerase. Segments 4-6 include HA, NP, and NA encoding virion proteins often subject to adaptive immune pressure. Segments 7-8 include M and NS.
(C) CpG content of IAV sequences from avian, human, and swine hosts segregated by common HA and NA subtypes.
(D) CpG content of IAV sequences segregated by time periods indicated on the x-axis (top). Schematic of IAV natural history showing avian H1N1, H2N1, and H3N2 in orange shaded lines, human circulating subtypes in shaded blue lines, 2009 H1N1 pandemic swine flu in green lines, (middle) and source of reassortant segments in matched colors (bottom).
Thick black bars represent median CpG content, boxes define the 25th to 75th percentile, and whiskers represent range excluding outliers. Horizontal dashed gray lines highlight median avian IAV CpG content as a reference.

Figure S2 Dinucleotide content of influenza viruses.
(A) CpG content as the normalized rho (ρ = #CpG / length divided by the product of #C / length and #G per length) of all IAV sequences from avian, human, and swine hosts as well as IBV and ICV.
(B) Percent GC content (%GC = (#G + #C) / total length) all IAV sequences from avian, human, and swine hosts as well as IBV and ICV.
(C) GpC content of all IAV sequences from avian, human, and swine hosts as well as IBV and ICV.
(D) Remaining dinucleotide content of all IAV sequences from avian, human, and swine hosts as well as IBV and ICV.

bioRxiv preprint doi: https://doi.org/10.1101/2024.12.23.629495; this version posted December 23, 2024. The copyright holder for this preprint (which was not certified by peer review) is the author/funder, who has granted bioRxiv a license to display the preprint in perpetuity. It is made available under aCC-BY 4.0 International license.

Figure S3 Structural comparison of avian ZAP proteins and data for avian ZAP knockdowns.

(A) Structure and model of human (blue; PDB: 6UEJ), chicken (RobettaCM, orange), chicken (RoseTTAFold, medium orange), and chicken (AlphaFold2, light orange) ZAP zinc fingers (1-4) with a CpGpU RNA oligonucleotide (gray; PDB: 6UEJ).

Structure and model of human (blue; PDB: 6UEJ), duck (RobettaCM, green), duck (RoseTTAFold, medium green), and duck (AlphaFold2, light green) ZAP zinc fingers (1-4) with a CpGpU RNA oligonucleotide (gray; PDB: 6UEJ).

Structure and model of human (blue; PDB: 6UEJ), quail (RobettaCM, pink), quail (RoseTTAFold, medium pink), and quail (AlphaFold2, light pink) ZAP zinc fingers (1-4) with a CpGpU RNA oligonucleotide (gray; PDB: 6UEJ).

Structure and model of human (blue; PDB: 6UEJ), chicken (RobettaCM, orange), duck (RobettaCM, green), and chicken (AlphaFold2, light pink) ZAP zinc fingers (1-4) with a CpGpU RNA oligonucleotide (gray; PDB: 6UEJ).

(B) Root mean squared deviation (RMSD) of structures and models in (A-D).

(C) qPCR results detecting endogenous gga *ZC3HAV1* mRNA in chicken DF-1 cells transduced with lentiviruses expressing shRNAs indicated (shades of gray) following treatment with 1000 U/mL of chicken interferon-alpha (IFN) relative to mock treated cells normalized to gga *TUBA1A*.

(D) Titers of PR8 and $PR8_{CG}$ over 48 hpi in chicken DF-1 cells stably expressing shRNAs targeting endogenous ggaZAP (shggaZAP1-3, shades of gray), a control scrambled sequence siRNA (shScramble, dark gray), and parental untransduced DF-1 cells (black). MOI=0.05.

(E) Titers of PR8 and $PR8_{CG}$ at 48 hpi in duck CCL-141 cells transiently transfected with 1nM or 10nM siRNAs targeting endogenous aplZAP (siZAP, shades of yellow), a control scrambled sequence siRNA (siNeg, 5nM), and parental untransfected CCL-141 cells. MOI=0.05.

Figure S4 TIDE analysis of pooled CRISPR/Cas9 chicken DF-1 and duck CCL-141 ZC3HAV1/ZAP knockouts.

(A-B) Schematic depicting exon organization of chicken ZC3HAV1/ZAP (orange) and duck ZC3HAV1/ZAP (yellow) as well as sites targeted by CRISPR single guide RNAs (sgRNAs; scissors). Colored boxes indicate coding exons and white boxes indicate noncoding portions of exons. Genomic sequence targeted by sgRNAs (underlined) and protospacer adjacent motif (PAM, lowercase) and amino acids encoding by that region (bold residues overlap sgRNA target). Genomic distances are relative and total locus size indicated.

bioRxiv preprint doi: https://doi.org/10.1101/2024.12.23.629495; this version posted December 23, 2024. The copyright holder for this preprint (which was not certified by peer review) is the author/funder, who has granted bioRxiv a license to display the preprint in perpetuity. It is made available under aCC-BY 4.0 International license.

(C) Total knockout efficiency as determined by tracking of indels by decomposition (TIDE) analysis (% Knockout) in each pool relative to LacZ control cells.

(D-F) TIDE analysis showing percent of sequences in pool exhibiting indicated indel (-10 to +10 bp from expected cut site) in chicken ZC3HAV1-targeted KO DF-1 cells.

(G-I) TIDE analysis showing percent of sequences in pool exhibiting indicated indel (-10 to +10 bp from expected cut site) in duck ZC3HAV1-targeted KO CCL-141 cells.

Red bars indicate disruptive indels (CRISPR-mediated insertions or deletions resulting in out-of-frame mutations). Gray bars indicate un-edited sequences. Black bars indicate in-frame indels (CRISPR-mediated insertions or deletions resulting in amino acid deletions).

Figure S5 Genetic and genomic comparisons of KHNYN genes

(A) Synteny diagrams of KHNYN genomic organization in representative species. KHNYN gene in blue, NYNRIN in magenta, neighboring human/pig/koala genes in black, neighboring platypus genes in red, and human/chicken genes syntenic to red genes in gray. Distances are relative and approximate size of locus shown on the right in kilobasepairs (Kbp).

(B) Schematic of exon organization (left) and protein domain architecture (right) of human N4BP1 (pink), KHNYN (blue), and NYNRIN (magenta). KH domain represents the extended HNRNPK-like, GXXG-like motif residues typical of KH-domains are indicated, NYN represents the NYN/PIN endonuclease domain, and CU represents cullin-binding domain associated with NEDD8 (KHNYN and NYNRIN) or cousin of CUBAN domain (N4BP1).

(C) Maximum likelihood phylogeny of all ensembl-annotated orthologues of human KHNYN, NYNRIN, and N4BP1.

(D) Synteny diagrams and schematics of exon organization for alligator, anole, and zebrafish *"khnyn-like"* genes.

(E) Structural predictions in three views of human (dark blue), pig (medium blue), dog (cyan), and platypus (lime green) KHNYN (residues 1-213). Putative GXXG motif highlighted by dashed black circle. Platypus-specific short loop highlighted by dashed black square.

Figure S6 Human transgenes do not affect IAV mRNA or chicken transcriptome.

(A) Principal component analysis (PCA) of RNA-seq data.

(B) Heatmap showing differentially expressed IAV mRNAs.

(C) Heatmap showing most differentially expressed chicken mRNAs.

Figure S7 Platypus KHNYN is a potent and broadly acting antiviral protein.

bioRxiv preprint doi: https://doi.org/10.1101/2024.12.23.629495; this version posted December 23, 2024. The copyright holder for this preprint (which was not certified by peer review) is the author/funder, who has granted bioRxiv a license to display the preprint in perpetuity. It is made available under aCC-BY 4.0 International license.

(A) Retroviral assembly and single-cycle infectivity results of mammalian ZAP-L and ZAP-S homologues including pig, dog, and platypus on ROSV infectivity. Platypus encodes a well annotated ZAP-L homologue but not a ZAP-S, a C653S prenylation mutant was included.
(B) Retroviral assembly and single-cycle infectivity results of mammalian KHNYN homologues including pig, dog, and platypus as well as catalytically inactivated (*"d"*) mutants of each on ROSV infectivity.
(C) Retroviral assembly and single-cycle infectivity results of platypus KHNYN including truncation mutants on ROSV infectivity (top). Schematic of protein domain architecture of platypus KHNYN with arrows indicating position of truncating stop mutation (bottom).
(D) Retroviral assembly and single-cycle infectivity results of platypus KHNYN including truncation mutants on ROSV infectivity (top). Indication (bottom) of virus tested (HIV-mCh, HIV_{CG}-mCh, or MLV-mCh) and glycoprotein used for pseudotyping (HIV-1 Env, VSV-G, or RABV-G).

Supplemental File 1. Key reagents. Spreadsheet
Supplemental File 2. Statistical analyses. Spreadsheet
Supplemental File 3. Influenza sequences, metadata, and R code. Spreadsheet, text files
Supplemental File 4. Human and avian ZAP structural models. Python files open in UCSC Chimera
Supplemental File 5. Human KHNYN, N4BP1, and HNRNPK structural models. Python files open in UCSC Chimera
Supplemental File 6. Mammalian KHNYN structural models. Python files open in UCSC Chimera
Supplemental File 7. N4BP1, KHNYN, and NYNRIN phylogenetic data. Clustal aln alignment, pdf of phylogenetic trees

Figure S1

bioRxiv preprint doi: https://doi.org/10.1101/2024.12.23.629495; this version posted December 23, 2024. The copyright holder for this preprint (which was not certified by peer review) is the author/funder, who has granted bioRxiv a license to display the preprint in perpetuity. It is made available under aCC-BY 4.0 International license.

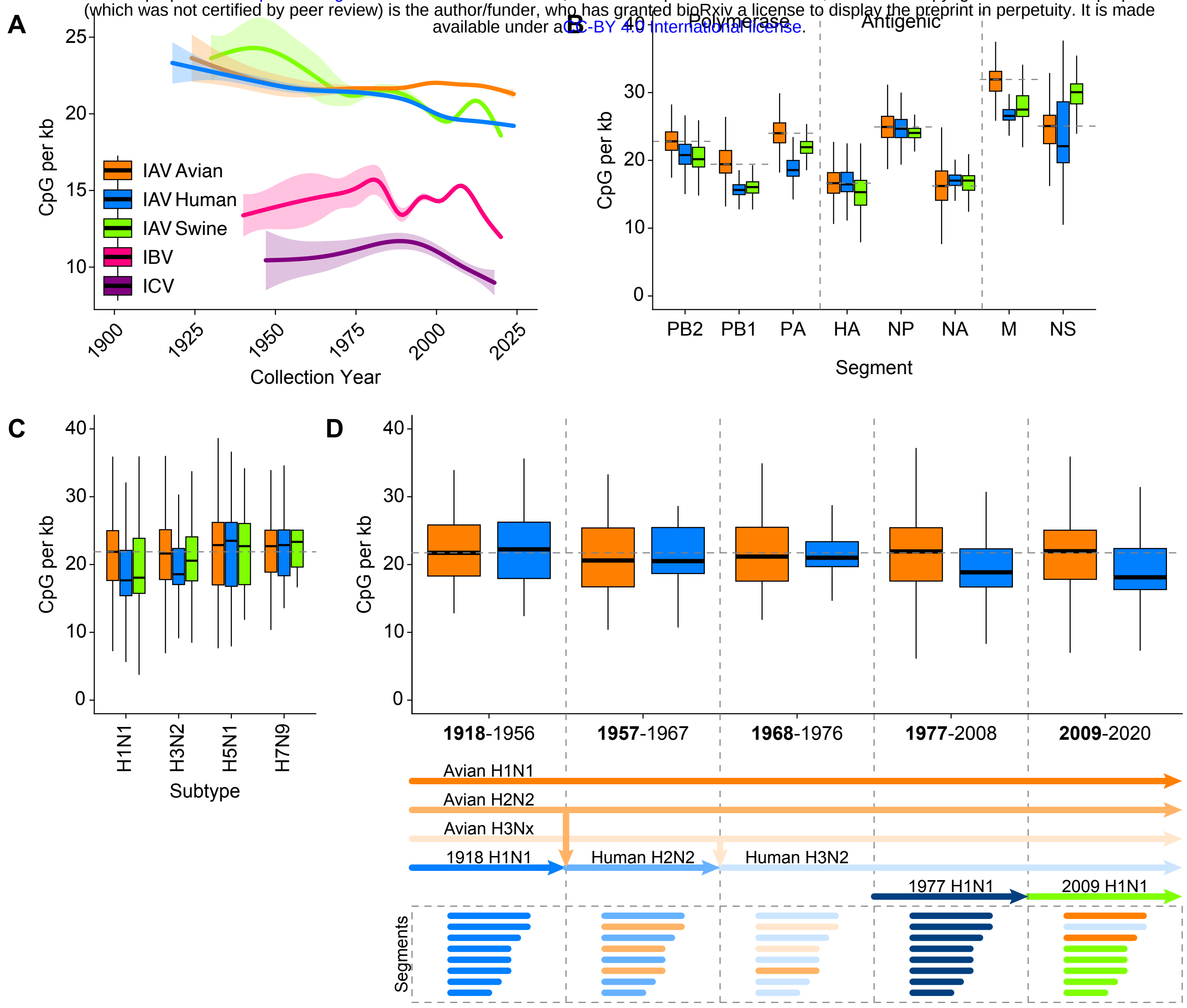

Figure S2

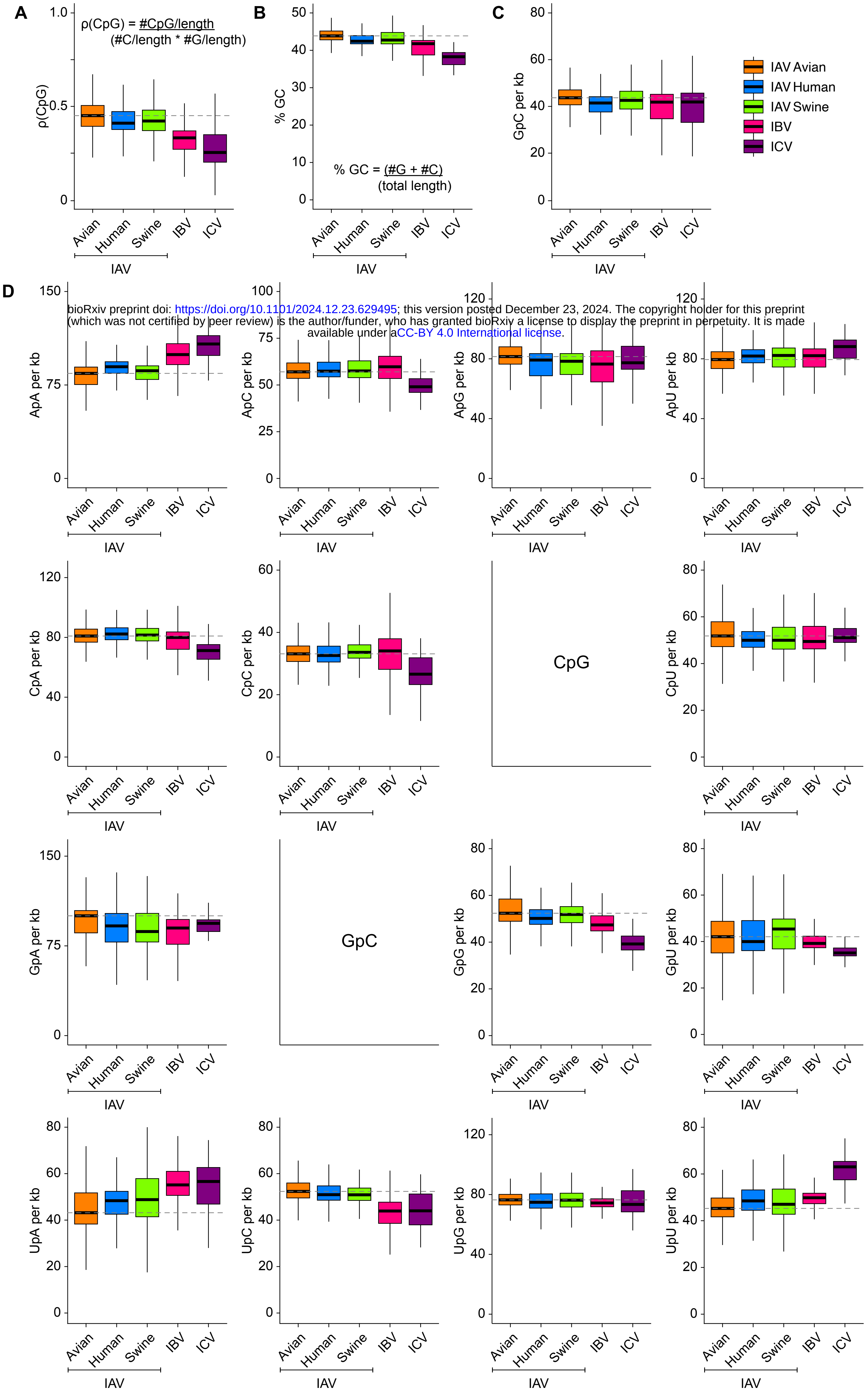

bioRxiv preprint doi: https://doi.org/10.1101/2024.12.23.629495; this version posted December 23, 2024. The copyright holder for this preprint (which was not certified by peer review) is the author/funder, who has granted bioRxiv a license to display the preprint in perpetuity. It is made available under aCC-BY 4.0 International license.

Figure S3

bioRxiv preprint doi: https://doi.org/10.1101/2024.12.23.629495; this version posted December 23, 2024. The copyright holder for this preprint (which was not certified by peer review) is the author/funder, who has granted bioRxiv a license to display the preprint in perpetuity. It is made available under aCC-BY 4.0 International license.

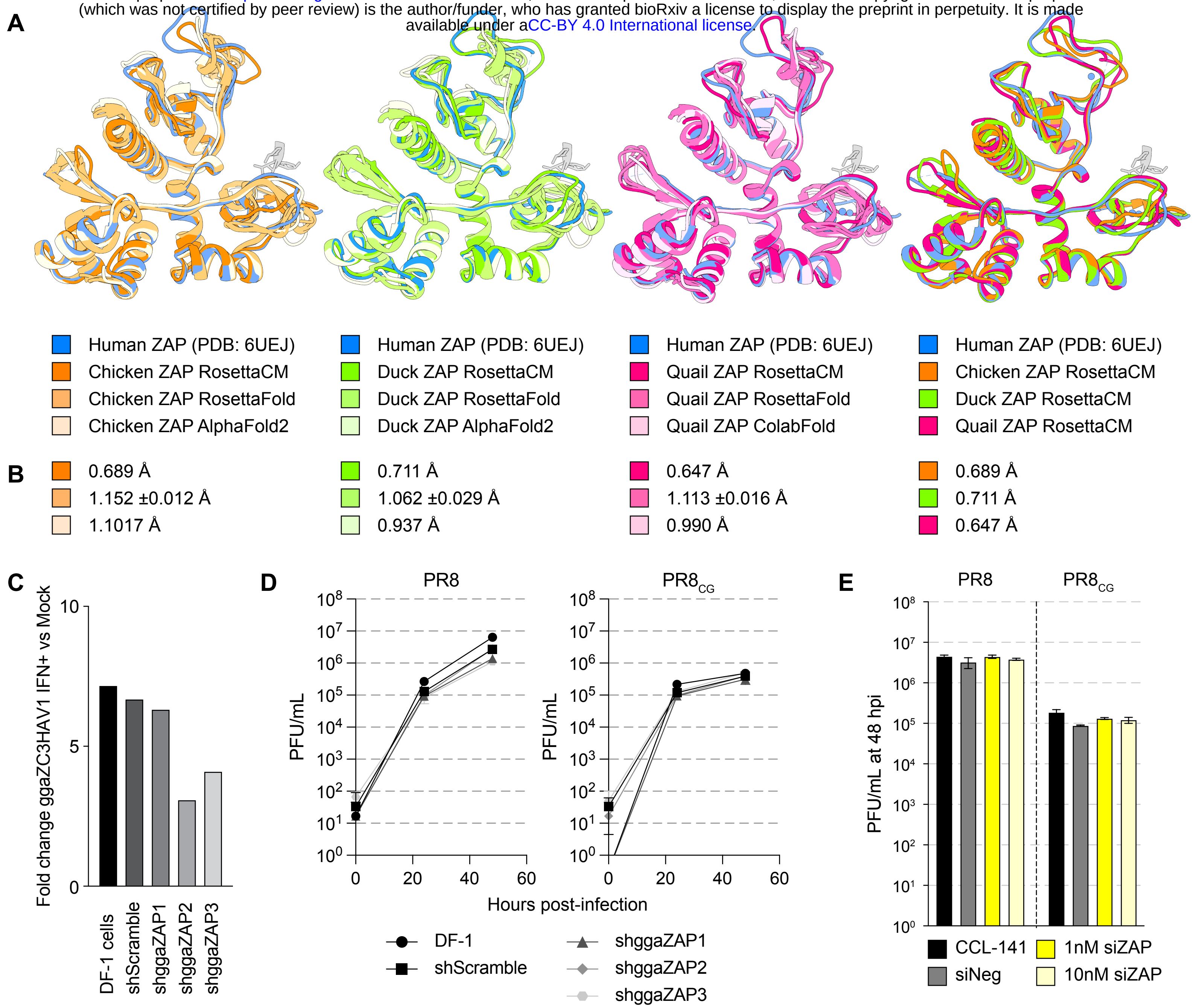

bioRxiv preprint doi: https://doi.org/10.1101/2024.12.23.629495; this version posted December 23, 2024. The copyright holder for this preprint (which was not certified by peer review) is the author/funder, who has granted bioRxiv a license to display the preprint in perpetuity. It is made available under aCC-BY 4.0 International license.

Figure S4

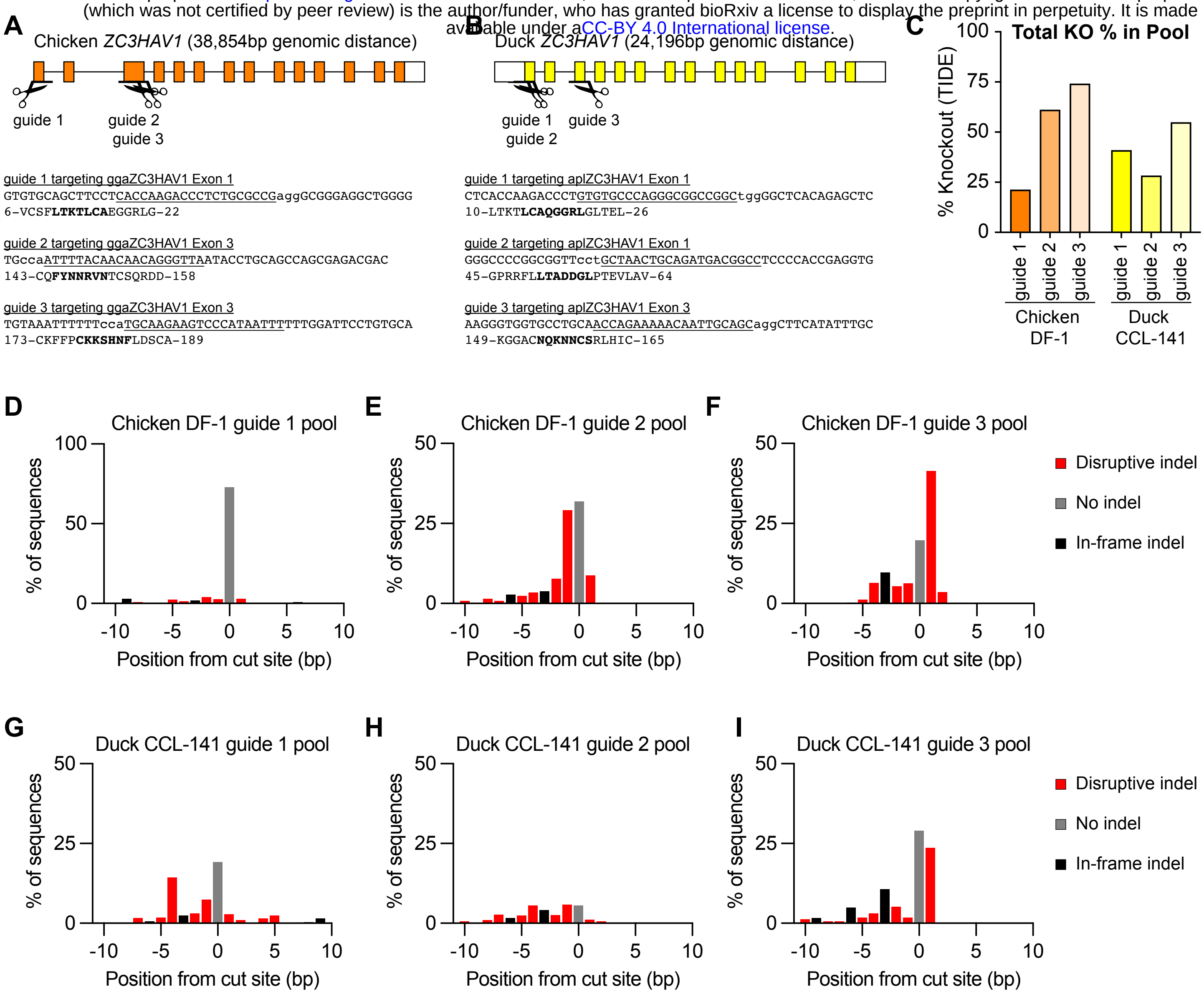

Figure S5

bioRxiv preprint doi: https://doi.org/10.1101/2024.12.23.629495; this version posted December 23, 2024. The copyright holder for this preprint (which was not certified by peer review) is the author/funder, who has granted bioRxiv a license to display the preprint in perpetuity. It is made available under aCC-BY 4.0 International license.

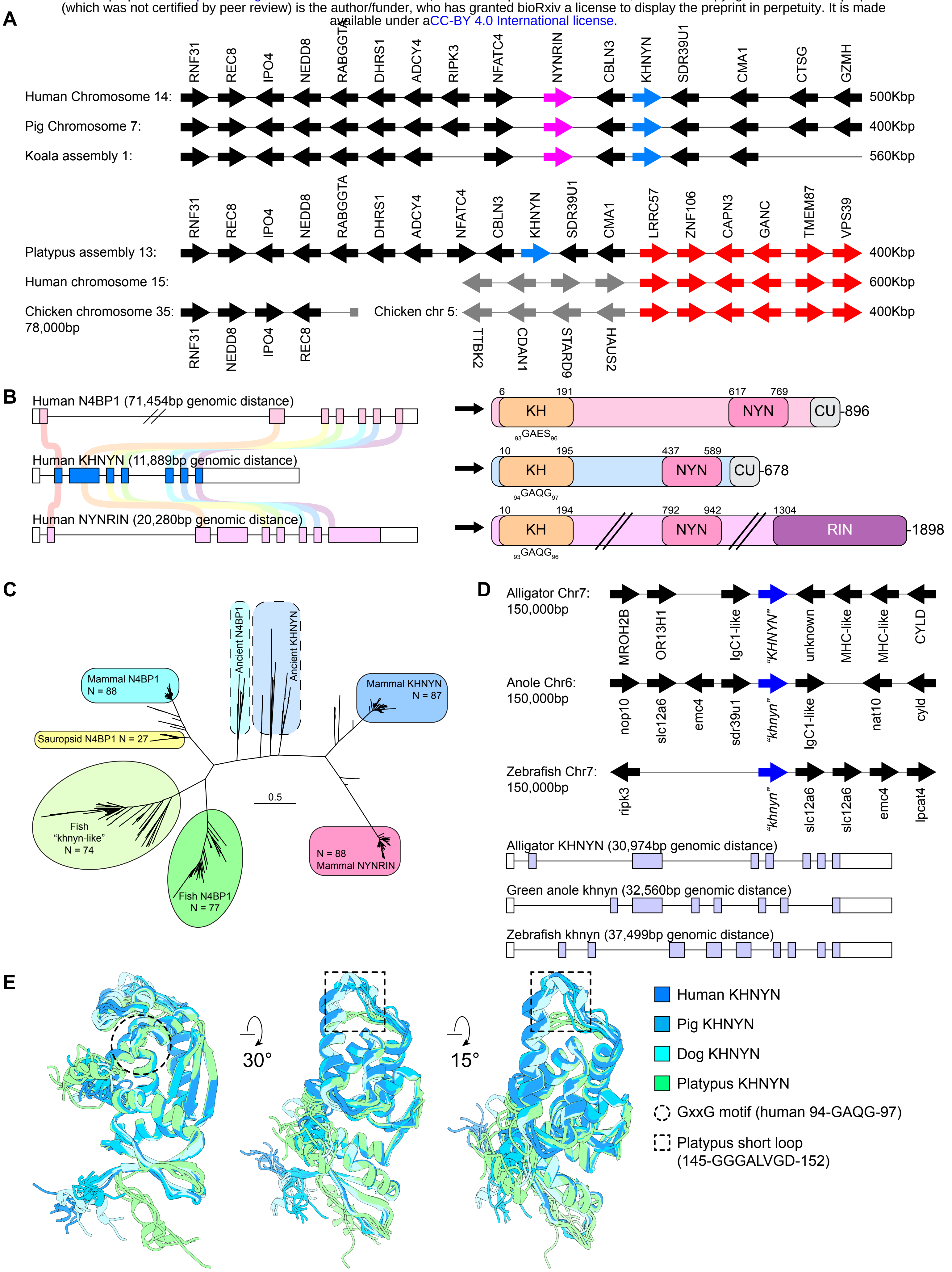

Figure S6

bioRxiv preprint doi: https://doi.org/10.1101/2024.12.23.629495; this version posted December 23, 2024. The copyright holder for this preprint (which was not certified by peer review) is the author/funder, who has granted bioRxiv a license to display the preprint in perpetuity. It is made available under aCC-BY 4.0 International license.

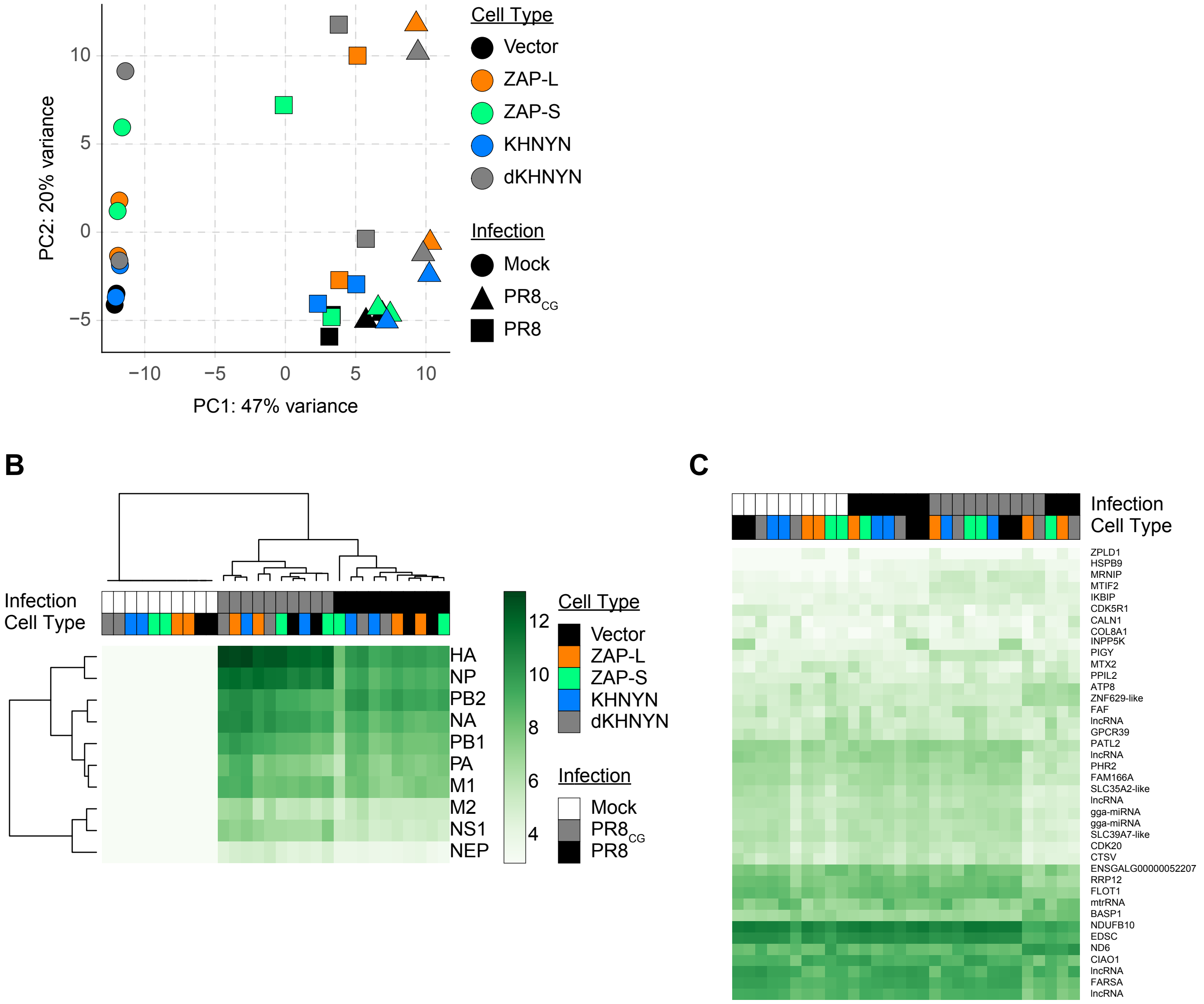

Figure S7

bioRxiv preprint doi: https://doi.org/10.1101/2024.12.23.629495; this version posted December 23, 2024. The copyright holder for this preprint (which was not certified by peer review) is the author/funder, who has granted bioRxiv a license to display the preprint in perpetuity. It is made available under aCC-BY 4.0 International license.

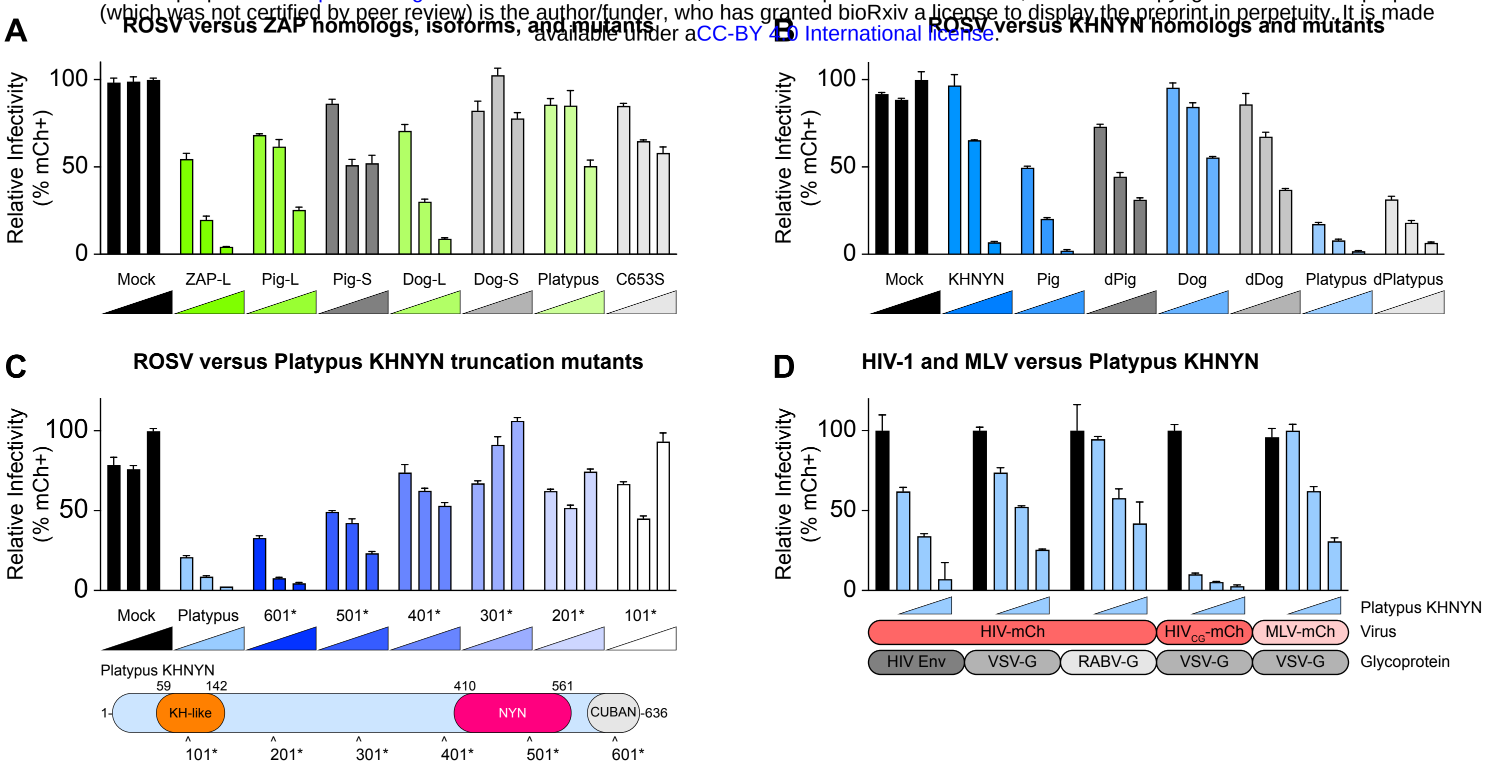

Supplement: Supplementary file 1 [file NIHPP2024.12.23.629495V1-supplement-1.pdf]
